# Distinct, dosage-sensitive requirements for the autism-associated factor CHD8 during cortical development

**DOI:** 10.1186/s13229-020-00409-3

**Published:** 2021-02-24

**Authors:** Shaun Hurley, Conor Mohan, Philipp Suetterlin, Robert Ellingford, Kimberley L. H. Riegman, Jacob Ellegood, Angela Caruso, Caterina Michetti, Olivier Brock, Romy Evans, Fabrizio Rudari, Alessio Delogu, Maria Luisa Scattoni, Jason P. Lerch, Cathy Fernandes, M. Albert Basson

**Affiliations:** 1 Centre for Craniofacial and Regenerative Biology, King’s College London, London, UK; 2 Department of Medical Biophysics, Mouse Imaging Centre, Hospital for Sick Children, University of Toronto, Toronto, ON Canada; 3 Department of Cell Biology and Neuroscience, Neurotoxicology and Neuroendocrinology Section, Istituto Superiore Di Sanità, Rome, Italy; 4 Department of Psychology, School of Behavioural Neuroscience, Sapienza University of Rome, Rome, Italy; 5 Centre for Synaptic Neuroscience and Technology, Istituto Italiano Di Tecnologia, Genova, Italy; 6 Department of Basic and Clinical Neuroscience, Institute of Psychiatry, Psychology and Neuroscience, King’s College London, London, UK; 7 MRC Social, Genetic and Developmental Psychiatry Centre, Institute of Psychiatry, Psychology and Neuroscience, King’s College London, London, UK; 8 MRC Centre for Neurodevelopmental Disorders, King’s College London, London, UK; 9 Present Address: Nuffield Department of Clinical Neurosciences, University of Oxford, Oxford, UK

**Keywords:** CHD8, Chromatin, TBR2, Autism, Cortex, Hypomorph, Conditional knockout, Mouse, Neural progenitor, Intermediate progenitor, p53, Gene expression, Proliferation, Apoptosis

## Abstract

**Background:**

*CHD8* haploinsufficiency causes autism and macrocephaly with high penetrance in the human population. *Chd8* heterozygous mice exhibit relatively subtle brain overgrowth and little gene expression changes in the embryonic neocortex. The purpose of this study was to generate new, sub-haploinsufficient *Chd8* mouse models to allow us to identify and study the functions of CHD8 during embryonic cortical development.

**Methods:**

To examine the possibility that certain phenotypes may only appear at sub-heterozygous *Chd8* levels in the mouse, we created an allelic series of *Chd8*-deficient mice to reduce CHD8 protein levels to approximately 35% (mild hypomorph), 10% (severe hypomorph) and 0% (neural-specific conditional knockout) of wildtype levels. We used RNA sequencing to compare transcriptional dysregulation, structural MRI and brain weight to investigate effects on brain size, and cell proliferation, differentiation and apoptosis markers in immunostaining assays to quantify changes in neural progenitor fate.

**Results:**

Mild *Chd8* hypomorphs displayed significant postnatal lethality, with surviving animals exhibiting more pronounced brain hyperplasia than heterozygotes. Over 2000 genes were dysregulated in mild hypomorphs, including autism-associated neurodevelopmental and cell cycle genes. We identify increased proliferation of non-ventricular zone TBR2+ intermediate progenitors as one potential cause of brain hyperplasia in these mutants. Severe *Chd8* hypomorphs displayed even greater transcriptional dysregulation, including evidence for p53 pathway upregulation. In contrast to mild hypomorphs, these mice displayed reduced brain size and increased apoptosis in the embryonic neocortex. Homozygous, conditional deletion of *Chd8* in early neuronal progenitors resulted in pronounced brain hypoplasia, partly caused by p53 target gene derepression and apoptosis in the embryonic neocortex.

Limitations

Our findings identify an important role for the autism-associated factor CHD8 in controlling the proliferation of intermediate progenitors in the mouse neocortex. We propose that CHD8 has a similar function in human brain development, but studies on human cells are required to confirm this. Because many of our mouse mutants with reduced CHD8 function die shortly after birth, it is not possible to fully determine to what extent reduced CHD8 function results in autism-associated behaviours in mice.

**Conclusions:**

Together, these findings identify important, dosage-sensitive functions for CHD8 in p53 pathway repression, neurodevelopmental gene expression and neural progenitor fate in the embryonic neocortex. We conclude that brain development is acutely sensitive to reduced CHD8 expression and that the varying sensitivities of different progenitor populations and cellular processes to CHD8 dosage result in non-linear effects on gene transcription and brain growth.

Shaun Hurley, Conor Mohan and Philipp Suetterlin have contributed equally to this work.

## Background

Mutations in *CHD8* (Chromodomain helicase DNA-binding protein 8) are some of the highest confidence risk factors for autism spectrum disorder (ASD) identified to date [1–5], with 96% of individuals with *CHD8* mutations presenting with autism, and 64% with macrocephaly [6, 7].

*CHD8* encodes a member of the ATP-dependent CHD chromatin remodelling family of proteins [8] and was initially identified as a direct repressor of β-catenin and p53 target genes [9–12]. Early embryonic lethality of homozygous *Chd8* deletion in the mouse is associated with p53-mediated apoptosis, consistent with its role as a transcriptional repressor of p53 target genes [11]. By contrast, CHD8 is typically recruited to promoters enriched for transcriptionally-permissive chromatin marks in neural progenitors, suggesting a role for CHD8 in transcriptional activation [13, 14]. Indeed, ASD-associated genes were downregulated in neural progenitor cells upon CHD8 knockdown [13, 14]. Evidence for mild brain overgrowth, reminiscent of the macrocephaly observed in patients with *CHD8* mutations, has been reported in several different *Chd8* heterozygous mouse models [15–18].

To explore the transcriptional dysregulation that may underlie abnormal brain development in heterozygous mice, gene expression has been investigated at different stages of brain development. These studies have revealed subtle gene expression changes in *Chd8*^+/−^ mice during embryonic development [15, 17–19]. By contrast, in vitro studies on neural progenitor cells have identified more substantial transcriptional dysregulation arising from CHD8 knock-down. For instance, Sugathan et al. observed 1756 differentially expressed genes (DEGs) upon *CHD8* knock-down in human iPSC-derived neural progenitor cells [14]. Gene expression changes in *Chd8*^+/−^ mice also appear to be strongly influenced by developmental stage, with more extensive transcriptional changes observed at peri- and postnatal stages [17, 18].

The striking phenotypes associated with human *CHD8* mutations and pronounced gene expression changes in neural progenitor cell lines upon CHD8 knockdown, contrast with the mild brain and embryonic transcriptional abnormalities observed in *Chd8* heterozygous mice. The only study so far to report convincing ASD-like behavioural phenotypes associated with *Chd8* deficiency, involved *Chd8* knock-down to ~ 20% of wildtype protein levels by selective in utero electroporation of neuronal progenitors contributing to upper layer neurons [20]. Together, these findings suggest that *CHD8* haploinsufficiency may have more pronounced effects on human brain development, or that some human ASD-associated mutations may reduce CHD8 function by more than 50%.

To examine the possibility that certain phenotypes may only appear at sub-heterozygous *Chd8* levels in the mouse, we created an allelic series of *Chd8*-deficient mice to reduce CHD8 protein gradually to approximately 35% (mild hypomorph), 10% (severe hypomorph) and 0% (conditional knockout) of wildtype levels. Non-monotonic effects on brain growth were observed, with mild hypomorphs exhibiting increased brain size, and severe hypomorphs and conditional knockout mice smaller brain size. We found that increased brain size in mild hypomorphs was associated with increased proliferation of TBR2+ intermediate progenitors. As this cell type contributes to increased human brain expansion during evolution [21], this finding suggests that CHD8 may have more pronounced effects on human brain development. We conclude that reducing CHD8 function below 50% has disproportionally large, and non-linear effects on gene expression and brain development.

## Methods

### Animals

**Fig. 1:**
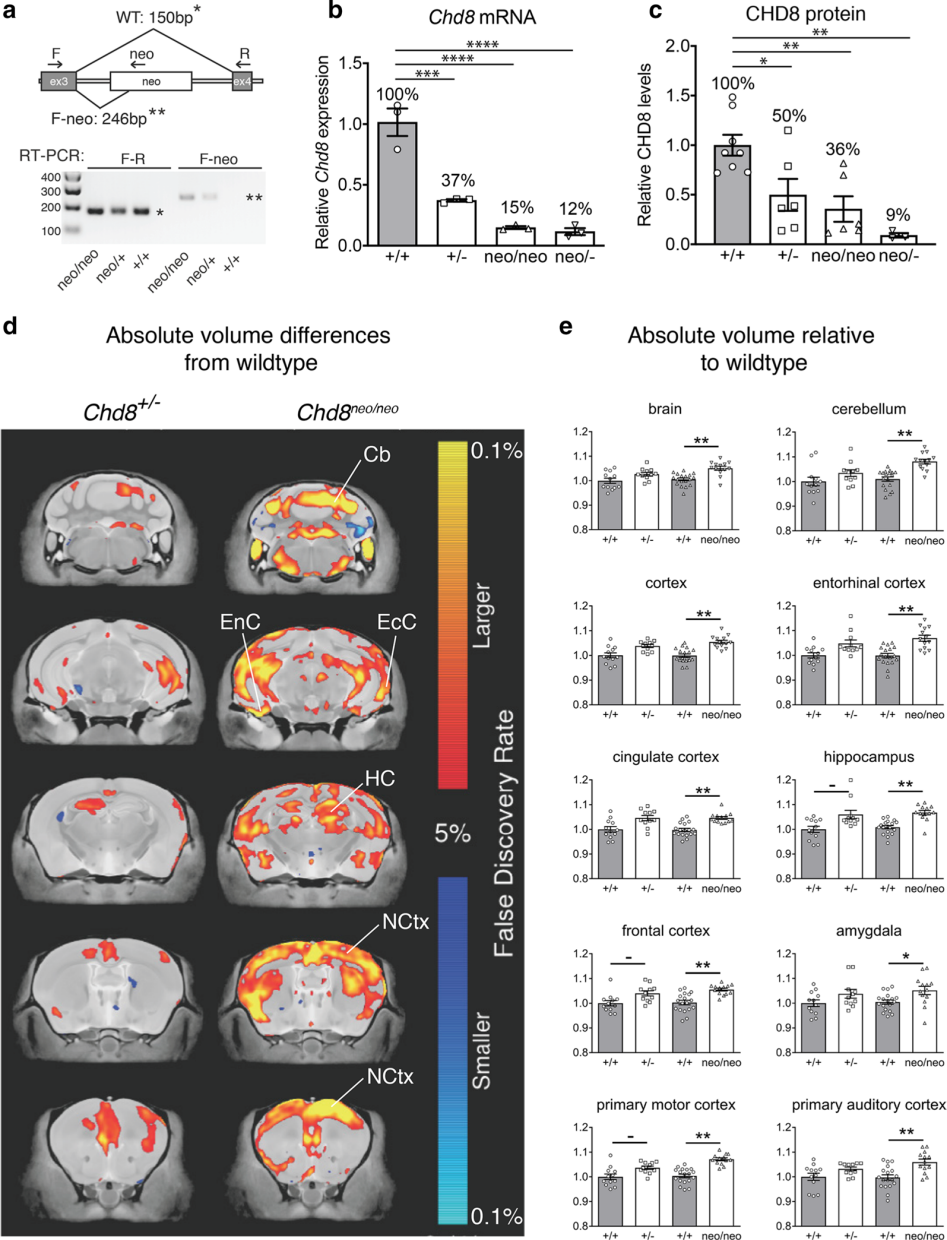
Brain hyperplasia in *Chd8* heterozygous and mild hypomorphic mice. **a** Diagrammatic representation of the *Chd8* allele containing the neo cassette between exons 3 and 4. Exon 3 splicing to exon 4 yields a 150 bp product (*) by RT-PCR using primers F and R. Aberrant splicing from exon 3 into the neo cassette yields a 246 bp product (**) with primers F and neo. **b** Quantitative RT-PCR of *Chd8* transcripts in E9.5-E10.5 neocortices of indicated genotypes. **c** Estimation of CHD8 protein levels in E12.5 neocortices by Western blot. *p < 0.05, **p < 0.01, ***p < 0.001.  **d** High-resolution 7 T structural MRI coronal images of *Chd8*^+/−^ (*n* = 12, all males, 22 weeks old) and *Chd8*^*neo/neo*^ brains (*n* = 13, 8 males, 5 females, 16 weeks old), from posterior (top) to anterior (bottom) are shown. Absolute volumetric differences, relative to wildtype controls (*n* = 30, 26 males, 4 females) are coloured according to the scale on the right. Some regions with enlarged volumes are labeled as follows: *NCtx* neocortex, *EcC* ectorhinal cortex, *EnC* entorhinal cortex, *HC* hippocampus, *Cb* cerebellum. **e** Absolute volumes relative to wildtypes are plotted for whole brain, neocortex and several other brain regions for the different genotypes as indicated. ^**–**^FDR < 0.15, *FDR < 0.05, **FDR < 0.01. See also Additional file 2: Table S1. MRI data from *Chd8*^+/−^ and littermate control mice used for comparison are from Suetterlin et al. [18]

A transgenic mouse line carrying a *Chd8*^*neo*^ allele (*Chd8*^*tm1.Mabn*^) was generated as reported previously [18]. Briefly, an 18.8 kb targeting construct was generated consisting of a 14.84 kb genomic DNA fragment subcloned from a C57BL/6 BAC clone (RP23:318M20) with an added loxP/FRT-PGK-gb2-Neo cassette 3′ of exon 3 (ingenious Targeting Laboratory (iTL), Ronkonkoma, NY, USA) and additional loxP site 5′ of exon 3 (Fig. 1). The targeting construct was linearised and electroporated in C57BL/6J ES cells. Five clones were identified with successful recombination, two of which (124 and 254) were injected into Balb/c blastocysts. Resulting chimaeras were backcrossed onto a C57BL/6J background to generate *Chd8*^*neo/*+^ mice. Experimental *Chd8*^*neo/neo*^ mice were produced by *Chd8*^*neo/*+^ × *Chd8*^*neo/*+^ crosses. To generate a conditional *Chd8* allele (*Chd8*^*flox*^ (*Chd8*^*tm1.1Mabn*^)), *Chd8*^*neo/*+^ mice were crossed with Flpe deleter mice on a C57BL/6J background (Fig. 5a). *Chd8*^*flox/*+^ mice were then either inter-crossed to obtain a homozygous *Chd8*^*flox/flox*^ line or with *Sox1-Cre* [22] to generate *Sox1-Cre; Chd8*^*flox/*+^ mice. To produce pan-neuronal *Chd8* null (conditional knockout, cKO) mice, *Sox1-Cre; Chd8*^*flox/*+^ mice were mated with *Chd8*^*flox/flox*^ mice. *Sox1-Cre; Chd8*^*flox/flox*^ cKO embryos were compared with *Sox1-Cre; Chd8*^*flox/*+^ (cHET) and *Chd8*^*flox/flox*^ (Ctrl) embryos. To generate conditional p53-heterozygotes, mice carrying the *Trp53*^*tm1Brn*^ conditional (*p53*^*flox*^) allele were obtained from the Jackson laboratories [23] and crossed to the *Chd8* conditional mice. *Chd8*^*flox/*+^ mice were also bred with *β-actinCre* mice [24] to generate a *Chd8* null (*Chd8*^*−*^*,* (*Chd8*^*tm1.2Mabn*^)) allele. *β-actinCre; Chd8*^+/−^ mice were then crossed with C57BL/6J mice to remove the Cre transgene and establish a *Chd8*^+/−^ line. *Chd8*^+/−^ mice were produced by *Chd8*^+/−^ × C57BL/6J crosses, taking care to equalise paternal or maternal inheritance of the *Chd8* null allele. Experimental *Chd8*^*neo/−*^ mice were produced by *Chd8*^*neo/*+^ × *Chd8*^+/−^ crosses. In experiments comparing different genotypes, replicate samples were from different litters to avoid litter-specific effects. All animal procedures were approved by the UK Home Office.

### Genotyping of mice

Genomic DNA was extracted for genotyping from ear notches (or yolk sac for embryos aged E14.5 and below) using Proteinase K digestion or the HotSHOT method [25]. Genotyping reactions were then performed for the presence of *Chd8* wildtype, neo, null or floxed alleles, p53 wildtype or floxed alleles, as well as the presence of Cre. Thermal cycles for all genotyping reactions were as follows: 94 °C, 5 min; 35X (94 °C, 30 s; 58 °C, 30 s; 72 °C, 30 s); 72 °C, 5 min. Primer pairs to amplify a sequence distinguishing between *Chd8*^*flox*^, *Chd8*^*neo*^ and wildtype alleles (‘*Chd8flox’* primers, 212 bp and 275 bp product for mutant or wildtype, respectively), to detect the presence of the *Chd8*^*null*^ allele (‘*Chd8null*’ primers, 395 bp), to distinguish between p53 floxed and wildtype alleles (‘*p53flox*’ primers, 390 bps and 270 bps, respectively) and primers to amplify a specific Cre sequence (‘*Cre*’ primers, 390 bp product) were used as listed in Additional file 1: Table S5.

### RNA extraction and qRT-PCR analysis

Cortical RNA was extracted by lysing cortices in 600 µl Trizol (Life Technologies). After purification, RNA was DNase treated using the Direct-zol RNA MiniPrep kit (Zymo Research) according to the manufacturer’s recommendations. cDNA was synthesised for qRT-PCR experiments using 50 ng RNA from 4 biological replicates per condition with the Precision nanoScript 2 Reverse Transcription Kit (PrimerDesign Ltd.) according to the manufacturer’s instructions. qRT-PCRs were performed on a Stratagene Mx3000p (Agilent Technologies) using PrecisionPlus-MX 2 × qPCR Mastermix with SYBR green (PrimerDesign Ltd.) and primers against *Atm, Atr, Trp53*, *Cdkn1a*, *Ccng1*, *Mdm2, Chd8, Pmaip1, Kdm5b, Zcwpw1* and *Stxbp1*. Relative expression levels were calculated using the 2^−∆∆CT^ method and *Gapdh* and *Ywhaz* were used as endogenous control genes.

### Western blot

Telencephalic vesicles were dissected from E12.5 embryos and whole cell protein prepared by lysing in 8 M urea, 1% CHAPS, 50 mM Tris (pH 7.9) lysis buffer containing protease inhibitors (PMSF, Pepstatin A, Leupeptin, Aprotinin; Roche) and a phosphatase inhibitor cocktail (Sigma). After rotating at 4 °C for 30 min, DNA was removed from lysates by centrifugation. Supernatant was transferred to a fresh tube and stored at -80 °C. Protein loading samples were made by diluting samples in Laemmli buffer containing 10% β-mercaptoethanol, followed by boiling at 100 °C for 10 min. Samples were loaded (10 µg total protein per lane) onto a Mini-PROTEAN pre-cast gel (Bio-Rad) and resolved using gel electrophoresis. Protein was transferred to a nitrocellulose membrane (Bio-Rad), which was then blocked in 5% non-fat milk powder (Bio-Rad) and 1% bovine serum albumin (BSA, Sigma) in TBS with 0.1% Tween-20 (TBST) for one hour at room temperature. Where β-actin was used as a loading control, the membrane was then cut in two: the higher molecular weight section was incubated with anti-CHD8 primary antibody (rabbit anti-CHD8 N-terminal, A301-224A, Bethyl Laboratories, 1/2000) and the lower molecular weight section incubated with anti-β-actin antibody (rabbit anti-β-actin, ab8227, Abcam, 1/4000); both antibodies in 3% non-fat milk powder and 1% BSA in TBST overnight at 4 °C. After washing, membrane was incubated with HRP-conjugated secondary antibody (Millipore) for one hour at room temperature. HRP was detected with Clarity ECL reagent (Bio-Rad) and the membrane imaged using a Bio-Rad ChemiDoc system. Where GAPDH was used as a loading control, the uncut membrane was washed in TBST after detection of CHD8 protein and incubated overnight at 4 °C in 0.05% sodium azide in PBS, before washing and incubation with anti-GAPDH primary antibody (rabbit anti-GAPDH, ab8245, Abcam, 1/40,000) overnight at 4 °C. Membrane was probed with HRP-conjugate and imaged as before. Raw proteins levels were quantified using Bio-Rad ImageLab software. All replicates on each given blot were first normalised to their respective GAPDH loading controls. Where the same samples were run across multiple blots their normalised values were averaged across all blots and this value used for statistical analysis. Normalised values for all samples were compared by ANOVA to identify statistically significant differences between the different genotypes.

### Structural MRI

Mice were terminally anaesthetised and intracardially perfused as described previously [18]. Voxelwise comparisons were made between mutants and all wildtypes taken from both the *Chd8*^+/−^ and *Chd8*^*neo/neo*^ batches. As wildtype brain sizes differed slightly between the two groups due to different age at analysis, *Chd8*^*neo/neo*^ data were first normalised (beta-corrected) to wildtypes in the *Chd8*^+/−^ batch before analysis. Voxelwise comparisons were then made between mutants and all wildtypes, and multiple comparisons in this study were controlled for using the False Discovery Rate [26].

### Behavioural assessments

Mice for behavioural testing were maintained as described previously [18]. Housing and test rooms were kept at constant temperature (21 °C) and humidity (45%) and maintained under a regular light/dark schedule with lights on from 07:30 to 19:30 h (light = 270 lx). All mice used in behavioural assessments were housed and tested at the same facility at King’s College London.

Different batches of *Chd8*^*neo/neo*^ mice were used for (i) recording pup ultrasonic vocalisations (USVs) and spontaneous movements, followed by tests for adult behaviours and (ii, iii) adult behaviours. For the first *Chd8*^*neo/neo*^ batch (i), tests were carried out in the following order: ultrasonic vocalisations (P2–P12), self-grooming (8–10 weeks), open field (9–11 weeks), adult social investigation (9–11 weeks), three-chamber social approach (9–11 weeks) and olfactory habituation/dishabituation (10–12 weeks). For the other two batches (ii, iii), tests were carried out as follows: rotarod (8–10 weeks), self-grooming (9–11 weeks), open field (10–12 weeks), adult social investigation (10–12 weeks), marble burying (11–13 weeks), 3 chamber social approach (11–13 weeks) and olfactory habituation/dishabituation (12–14 weeks). The final batch was then further assessed using the running-wheel test (16–20 weeks). Data from different batches did not differ significantly and were all combined for analysis.

One week before performing the rotarod test, mice were singly-housed to avoid any potential confounds from social and aggressive behaviour hierarchies, which could influence the controlled assessment of social behaviours [27]. Sawdust was changed every other week but never on the day before, or the day of testing. The enrichment (nesting material and house) was changed less regularly to minimize the disruption to the animals. For all social tests, conspecific mice were housed in a separate room to the test mice to ensure the conspecifics were unfamiliar to the test mice. Test mice were never exposed to the same conspecific during testing to ensure novelty.

Behavioural experiments were conducted between 08:30 and 18:30 in sound-proofed rooms under standard room lighting unless stated otherwise. Behaviours were recorded using a camera positioned above the test arenas and movement of each mouse tracked using EthoVision (Noldus Information Technologies, bv Wageningen, The Netherlands). Social investigation, olfaction and grooming were scored manually using MATLAB 2016b (The MathWorks, Inc., Natick, Massachusetts, USA). After each individual trial of a specific test, boli and urine were removed from the test arena which was cleaned with 1% Anistel® solution (high level surface disinfectant, Trisel Solution Ltd, Cambridgeshire, UK) to remove any odours. Experimenters were blind to the genotype of the animals both during the testing and subsequent scoring of the recorded behaviours.

### RNA sequencing

For RNA-sequencing at E10.5, total RNA from 2 embryos was pooled for each biological replicate (*n* = 3 per condition). No pooling was performed at E12.5 (*n* = 3 per condition). mRNA was isolated and reverse transcribed into cDNA. cDNA was end-repaired, adaptor-ligated and A-tailed. Paired-end sequencing (75 bp read length) was performed on the Illumina HiSeq 4000 platform. Quality of the raw sequencing data was checked using FastQC version 0.11.2 and trimming of adaptor sequences was performed using Trim Galore! version 0.4.1 [28]. Reads were aligned to the mouse genome (GRCm38.p4) using Tophat version 2.1.0 and aligned reads were counted using FeatureCounts version 1.5.0 [29, 30]. Differential expression testing was performed using DESeq2 version 1.10.1, as previously described [31]. Gene ontology analysis and functional classification was performed using DAVID with all detected DEGs below a 0.05 FDR [32]. For heatmaps, data were transformed with a variance stabilising transformation, scaled and clustered with the Ward.d2 method using maximum distance, and plotted with the R package pheatmap version 1.0.8. The R package ggplot2 version 2.1.0 was used to generate volcano plots and DESeq2 was used to generate normalised read count plots for individual genes. The list of ASD associated genes used for overlap with the hypomorph DEGs was obtained from the SFARI Human Gene database (https://gene.sfari.org/autdb/HG_Home.do). RNA-seq data have been deposited into GEO, accession number GSE121381.

### Tissue collection and processing

Embryos were collected and brains dissected from the skulls in ice-cold PBS for E18.5 embryos. Wholemount pictures were taken on a Nikon SMZ1500 stereomicroscope equipped with a Nikon DS-Fi1 camera head, followed by fixation in 4% PFA for 24 h at 4 °C. For BrdU experiments, pregnant mothers (day 13 or 15 of gestation) were injected with 40 mg/kg BrdU in 0.9% saline 1 h prior to embryo collection. After fixing, embryos were dehydrated and paraffin embedded. Paraffin blocks were then cut into 10 µm (cKO and cKO; p53-het embryos) or 5 µm (*Chd8*^+/−^*, Chd8*^*neo/neo*^ and *Chd8*^*neo/−*^ embryos) thick coronal sections and mounted on slides.

### X-gal staining

E9.5 embryos were collected and dissected in ice-cold PBS and fixed in 4% PFA for 10 min. Following three washes in PBS (5 min each), embryos were incubated in X-Gal staining solution (10 mM TRIS–HCL, pH 7.3, 0.005% Na-deoxycholate, 0.01% IGEPAL, 5 mM potassium ferrocyanide, 5 mM potassium ferricyanide, 2 mM MgCl_2_, 0.8 mg/ml X-Gal, in PBS) at room temperature until adequate signal was observed. Reactions were stopped by washing in PBS (3 × 5 min) followed by post-fixation in 4% PFA for 1 h. Control embryos never showed any staining.

### Immunohistochemistry and immunofluorescence

Coronal brain sections were re-hydrated using standard protocols. Antigen retrieval was conducted by heating slides in 10 mM Sodium Citrate solution (pH6) for 20 min and cooled on ice. For non-fluorescence immunohistochemistry, endogenous peroxidases were blocked by incubating in 3% H_2_O_2_ and 10% MeOH in PBS for 15 min. Sections were then washed in 0.2% Triton X-100 (Sigma-Aldrich) in PBS (PBT2) for 5 min and blocked using 10% heat-inactivated normal goat serum (GS) and 2% gelatin in PBT2 for 1 h. Sections were incubated in 5% GS in PBT2 containing primary antibody overnight at 4 °C. The following antibodies were used: mouse anti-BrdU (BD Biosciences, 1/100), rabbit anti-phosphohistone 3B (Cell Signaling, 1/100), mouse anti-phosphohistone 3B (Abcam, 1/200), chicken anti-TBR2 (Merck Millipore, 1/200), rabbit anti-cleaved-caspase 3 (Cell Signaling, 1/200), rabbit anti-doublecortin (Abcam, 1/400) or rabbit anti-CHD8 (Bethyl, 225A, 1/400). For immunofluorescence, sections were incubated with secondary antibody diluted in 5% GS in PBT2 for 90 min at 4 °C. Secondary antibodies used included goat anti-chicken AlexaFluor 488 (Invitrogen, 1/200), goat anti-mouse AlexaFluor 405 (Invitrogen, 1/200), goat anti-mouse AlexaFluor 594 (Invitrogen, 1/200), goat anti-rabbit AlexaFluor 488 (Invitrogen 1/200), goat anti-rabbit AlexFluor 568 (Invitrogen, 1/200) and donkey anti-rabbit AlexaFluor 488 (Invitrogen, 1/200). Sections were counterstained using Hoechst 33342 solution (Invitrogen, 1/50,000) in PBS and covered with coverslips using CitiFluor (CitiFluor Ltd., UK). For diaminobenzidine (DAB) immunohistochemistry, after incubation with primary antibody, sections were incubated in biotinylated anti-rabbit immunoglobulin secondary antibody (Dako, 1/200) in 5% GS in PBT2. Samples were washed in PBS and incubated with Avidin/biotin complex (ABC, Vector) in PBS for 1 h. Sections were developed using 0.03% DAB and 0.0003% H_2_O_2_ in PBS for 10 min before washing in running water and counterstaining using Ehrlich’s Haemotoxylin solution. Slides were mounted onto coverslips using DPX (Sigma-Aldrich). Images were acquired on a Nikon 80i microscope equipped with a Hamamatsu C4742 CCD or Nikon 5 M pixel Nikon DS digital cameras. Images were processed using Adobe Photoshop and Illustrator.

### Fluorescence-activated cell sorting (FACS) and qRT-PCR

Pregnant females were sacrificed by cervical dislocation on embryonic day 14.5 (E14.5) and embryos were dissected out and placed in ice cold Hibernate-E solution supplemented with B27. The brain was dissected out from each embryo and the cortex removed. Cortices were maintained in ice-cold Hibernate-E/B27 whilst embryos were genotyped, after which cortices with corresponding genotypes were pooled together in digest solution (0.25% Trypsin, 0.01% DNase, 10 mM HEPES in HBSS) preheated to 37 °C. Cortices were incubated in digest solution for 10 min and washed in HBSS + 0.01% DNase to inactivate trypsin. Single-cell suspension was achieved through mechanical dissociation by gentle pipetting. Cells were washed and resuspended with PBS, incubated on ice and in the dark for 30 min with 1 µl of the near-IR fluorescent reactive dye from the LIVE/DEAD fixable dead cell stain kit (ThermoFisher). Cells were washed once with PBS, strained into fixing solution (1.6% PFA and 0.1% saponin in molecular-grade PBS) supplemented with 1% RNasin Plus RNAse inhibitor and incubated on ice for 30 min. Cells were washed twice with wash solution (0.2% BSA, 0.1% saponin, 0.1% RNasin Plus in molecular-grade PBS) before antibody staining. The following primary antibodies were used: phycoerythrin (PE)-conjugated mouse anti-PAX6 (sc-81649, Santa Cruz Biotechnology) and chicken anti-TBR2 (AB15894, Merck Millipore). Cells were suspended in staining solution (1% BSA, 0.1% saponin, 1% RNasin Plus in molecular-grade PBS) containing primary antibodies at a 1:1000 dilution and incubated on ice for 30 min. Cells were washed twice with wash solution then suspended in staining solution containing anti-chicken AlexaFluor 488 secondary antibody (A-11039, ThermoFisher) at a 1:1000 dilution. Cells were washed twice with wash solution then strained and resuspended in sorting buffer (3% BSA and 2 mM EDTA in molecular grade PBS). Fluorescence-activated cell sorting (FACS) was performed using a FACSAria III machine (BD). Samples stained individually for TBR2 and PAX6 were used as compensation controls. Debris was excluded from samples by gating on a forward and side scatter area plot and doublets excluded by gating on a plot of side scatter area by side scatter width. The LIVE/DEAD marker was detected in the APC-Cy7 channel. Any cells showing greater fluorescence intensity than an unstained control sample were considered dead and excluded. The unstained sample was also used to set a double negative gate on a PE by FITC fluorescence intensity plot. The PAX6+ gate was placed based on increased PE fluorescence and the TBR2+ gate placed based on increased FITC fluorescence. Selection gates were placed towards the extreme ends of the cell population to ensure the sorting of single positive cells only. Cells falling within the PAX6+ or TBR2+ gates were sorted and collected in sorting buffer. Cells were then pelleted, snap frozen in liquid nitrogen and stored at −80 °C. RNA was extracted from sorted cell samples using the RecoverAll Total Nucleic Acid isolation kit (Life technologies) using the RNA isolation protocol with an adapted protease treatment for 3 h at 50 °C to maximise RNA yield. RNA concentration was determined with a NanoDrop 2000 (ThermoFisher). Typically, samples yielded approximately 120 ng of RNA which was stored at −80 °C. Reverse transcription was performed using the Precision nanoScript2 Reverse Transcription Kit (Primerdesign) according to the manufacturer’s instructions using random nonamer primers. 60 ng of RNA was reverse transcribed for all samples and resulting complementary DNA (cDNA) stored at −20 °C. qPCRs were performed using Luna Universal qPCR Master Mix (New England Biolabs). Samples of 3.75 ng cDNA were run in triplicate for each primer pair using a Lightcycler 480 (Roche) and fold expression changes were calculated using the 2^−ΔΔCT^ method. Primer sequences are given in Additional file 1: Table S5.

## Quantification and statistical analysis

### General

Data are reported as Mean ± SEM and graphs show all individual data points where feasible. Significant *p* values are reported in the results section and figure legends provide details of relevant statistical parameters, including group sizes. Statistical analyses were performed either with SPSS (Version 22, IBM, Armonk, USA) or GraphPad Prism (Version 6, GraphPad Software, La Jolla, California, USA). All analyses were performed blind to genotype.

### RNAseq

Processing of raw data and differential expression testing is described in the methods section. Multiple comparisons were controlled for using an FDR < 0.05. Exact *p* values and FDR adjusted *p* values for all differentially expressed genes are listed in Additional file 3: Tables S2 and Additional file 5: Table S4.

### Cell marker quantification

#### Proliferation

Proliferation was quantified by counting either ventricular or non-ventricular phosphohistone 3B-positive cells and normalising cell counts to the length of ventricular surface. Cells were counted in sections located between A-P positions 219–236 of the E14.5 Allen brain reference atlas for coronal sections (https://developingmouse.brain-map.org/experiment/thumbnails/100074513?image_type=hp_yellow) and from the boundary with the eminences up to the "apex" of the neocortex in the M-L axis. Normalised counts were averaged across both hemispheres for at least three sections to give the number of phosphohistone 3B-positive cells per μm of ventricular surface in the dorsal cortex of each embryo. To determine the molecular identity of non-ventricular PH3B+ cells at E14.5, sections were co-stained for TBR2 and the number of TBR2/PH3B double-positive cells counted and normalised to ventricular surface length.

#### Apoptosis

Cleaved Caspase 3 (CC3) positive cells were counted in the E14.5 neocortex and normalised to ventricular zone length in each hemisphere as described above. Counts were averaged across both hemispheres and across a minimum of three sections per biological replicate. In E12.5 cKO embryos, CC3-positive cells were counted in 50 μm × 50 μm boxes. Three boxes were counted for both inner (ventricular side) and outer (pial side) regions of the dorsal cortex to generate an average number of CC3-positive cells per μm^2^ for both inner and outer cortical regions, which were then averaged to provide the overall mean of CC3-positive cells per μm^2^. These were calculated for both sides of the brain individually in at least two sections per biological replicate. Apoptosis in the ventral cortex was quantified by counting CC3+ cells either in three 0.1 mm × 0.1 mm boxes in both lateral ganglionic eminences and three 0.15 mm × 0.15 mm boxes in both medial ganglionic eminences (WT and cHet), or three 0.1 mm × 0.1 mm boxes placed at equivalent positions across the ventral cortex (cKO and cKO-p53Het) and counts averaged for each section. These were calculated for both sides of the brain individually in at least two sections per biological replicate.

### Data availability

The RNAseq raw data and read counts were deposited at the Gene Expression Omnibus (GEO) archive under the Accession Number GSE121381.

## Results

### A *Chd8* allelic series reveals non-linear effects of CHD8 deficiency on brain growth

To establish a *Chd8* allelic series in the mouse, we first generated a hypomorphic *Chd8* allele (*Chd8*^*neo*^, Fig. 1a, Fig. 5a) by inserting a neo cassette between exons 3 and 4 to reduce gene expression through splicing and termination of transcripts [33, 34]. Aberrant splicing of *Chd8* transcripts into the neo cassette was confirmed (Fig. 1a). *Chd8*^*neo/neo*^ and *Chd8*^*neo/−*^ embryos showed 85% and 88% reductions in *Chd8* transcripts, respectively, compared to the 63% decrease in *Chd8*^+/−^ embryos (Fig. 1b). Full-length CHD8 protein levels were reduced by approximately 50% in *Chd8*^+/−^ (heterozygous)*,* 64% in *Chd8*^*neo/neo*^ (mild hypomorph), and 91% in *Chd8*^*neo/−*^ (severe hypomorph) neocortices, with no evidence for remaining truncated CHD8 protein products (Fig. 1c, Additional file 1: Fig. S1).

*Chd8*^*neo/neo*^ mice exhibited a significant reduction in postnatal survival (Table 1). As CHD8 is expressed in multiple tissues during development [35], this postnatal lethality is likely a consequence of congenital defects affecting essential organs.

**Table 1:** Reduced postnatal survival of *Chd8*^*neo/neo*^ pups

	Births	P0	P14***	P35***	Expected %
*Chd8*^*neo/neo*^	24.7	16.2	**8.1**	**8.2**	25
*Chd8*^*neo/*+^	51.7	56.8	59	55.7	50
*Chd8*^+*/*+^	23.6	27	32.8	36.1	25
	*n* = 89	*n* = 74	*n* = 271	*n* = 97	

Bold indicates significant deviation from expected frequencies

Mice were born at expected Mendelian frequencies from *Chd8*^*neo/*+^ intercrosses. Percentage survival is shown for the different genotypes at P0, P14 and P35. Note the significantly reduced percentage of homozygous *Chd8*^*neo/neo*^ mutants obtained at P14 and P35 (***p < 0.001, Chi-square test) in two independent groups of mice.

High resolution structural MRI revealed volumetric increases in a number of brain regions in *Chd8*^*neo/neo*^ mice compared to wildtype littermates (Fig. 1d). This phenotype was more pronounced than in *Chd8*^+/−^ mice (Fig. 1d), with total brain volume increased by 4.5% in *Chd8*^*neo/neo*^ mice, compared to the 2.7% increase in *Chd8*^+/−^ mice (Fig. 1e). As our *Chd8*^+/−^ data were from males only, we also calculated the increase in brain volume increase in male *Chd8*^*neo/neo*^ mice only, to exclude the possibility that female *Chd8*^*neo/neo*^ mice were responsible for the observed increase in brain volume. The average brain volume of *Chd8*^*neo/neo*^ male mice (*n* = 8) were increased by 5.4% compared to their male wildtype littermates (*n* = 14), compared to the 2.7% increase in volume of male *Chd8*^+/−^ mice (*n* = 12) compared to their male wildtype littermates (*n* = 12). Several regions that showed evidence of overgrowth in *Chd8*^+/−^ mice (Fig. 1e, Table 2) demonstrated robust increases in volume in *Chd8*^*neo/neo*^ mice, including the frontal, cingulate and entorhinal cortices and the hippocampus (Fig. 1e, Table 2, Additional file 2: Table S1).

**Table 2:** Brain volume differences relative to control wildtype littermates

Brain area	*Chd8* genotype	
	+/− (%)	neo/neo (%)
Brain volume	2.73	4.53**
Cortex	3.82^−^	5.53**
Cerebellum	3.41	6.99**
Hippocampus	5.97^−^	5.84**
Primary motor cortex	3.68^−^	6.68**
Primary somatosensory cortex	3.7^−^	5.62**
Primary auditory cortex	3.2	6.23**
Primary visual cortex	3.39	7.15**
Frontal cortex	3.99^−^	5.21**
Frontal association cortex	4.81^−^	6.07**
Entorhinal cortex	4.83^−^	7.05**
Cingulate cortex	4.57^−^	4.84**
Amygdala	3.82	4.64*

^−^FDR < 0.15; *FDR < 0.05; **FDR < 0.01. MRI data from *Chd8*^+/−^ and littermate control mice used for comparison are from Suetterlin et al. [18]

To determine the effects of reducing *Chd8* levels further, we assessed postnatal survival of *Chd8*^*neo/−*^ severe hypomorphs. *Chd8*^+/−^  × *Chd8*^*neo/*+^ matings yielded no live *Chd8*^*neo/−*^ animals by P7–P14 (*n* = 0/22), indicative of a significant postnatal lethality (*p* < 0.001; Chi-square test). To assess the phenotype of severe hypomorphs, we collected embryos at E18.5. Measuring brain weights in these embryos, revealed that *Chd8*^*neo/−*^ brains were 13.4 mg (18.3%) lighter than brains from *Chd8*^+*/*+^ littermates (Additional file 1: Fig. S2A, B, E, G). *Chd8*^+/−^ brains weighed on average 4.74 mg (6.47%) heavier than controls (Additional file 1: Fig. S2C, D, F, G), in agreement with our previous report [18]. In addition, 50% of *Chd8*^*neo/−*^ embryos showed unilateral anopthalmia (Additional file 1: Fig. S2H), in agreement with strong *Chd8* expression in the developing eye [35]. Taken together, these data suggested that brain growth responds to reductions in CHD8 levels in a non-linear manner, with heterozygotes and mild hypomorphs exhibiting brain overgrowth, and severe hypomorphs presenting with brain hypoplasia.

### *Chd8*^neo/neo^ mice demonstrate mild behavioural anomalies similar to those seen in *Chd8*^+/*−*^ mice

**Fig. 2:**
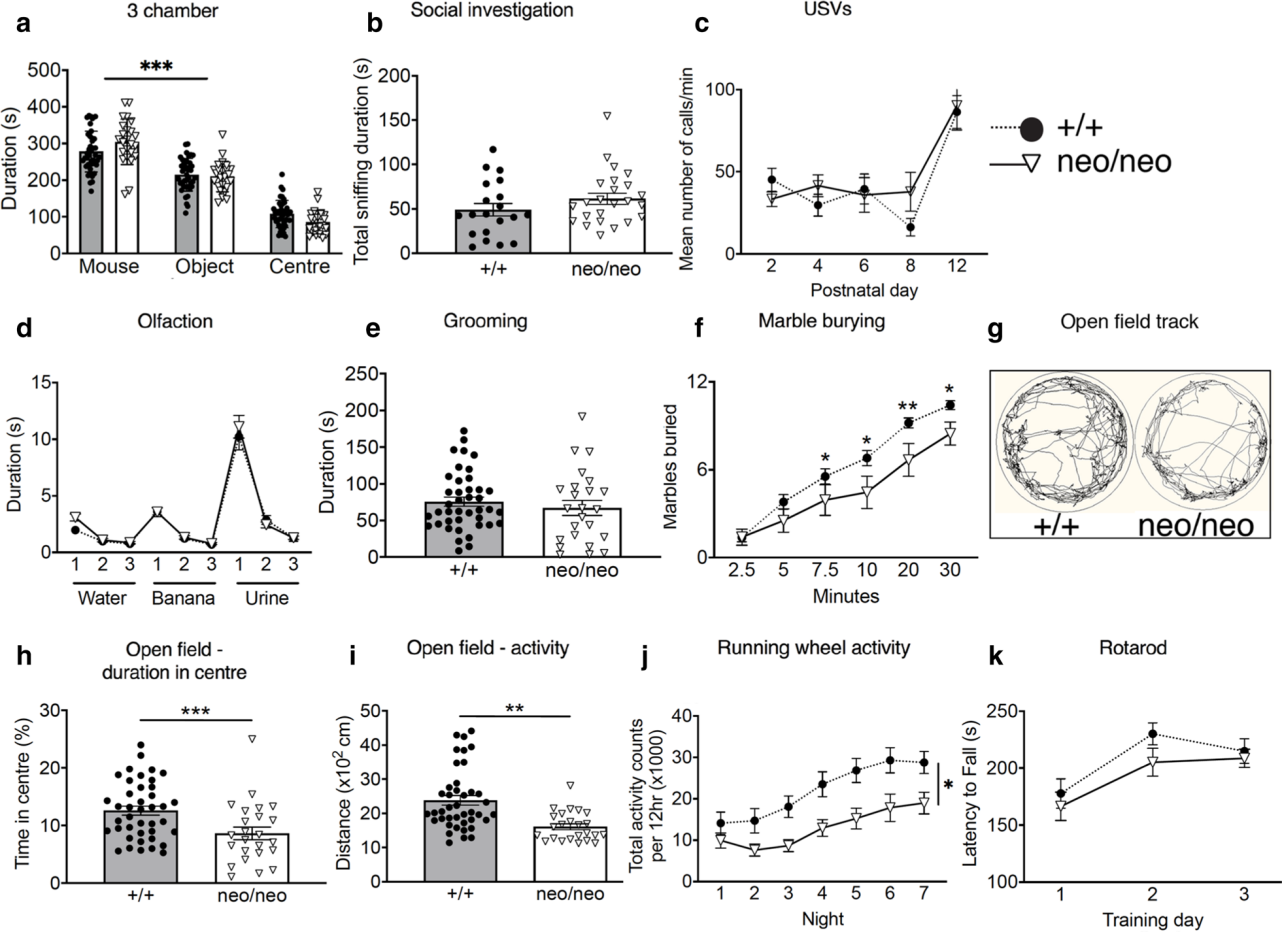
Behavioural assessment of mild *Chd8* hypomorphic mice. **a**–**k** Behavioural assessments of a cohort of adult *Chd8*^*neo/neo*^ (neo/neo, *n* = 24; 14 males, 10 females, **a**, **b**, **d**, **e**, **g**, **i** or *n* = 15; 10 males, 5 females, **f**, **k** or *n* = 11; 6 males, 5 females, **j**) and *Chd8*^+*/*+^ (+/+, *n* = 41; 26 males, 15 females, **a**, **b**, **d**, **e**, **g**, **i** or *n* = 31, 21 males, 10 females, **f**, **k** or *n* = 12; 6 males, 6 females, **j**) and pup *Chd8*^*neo/neo*^ (neo/neo, *n* = 10; 4 males, 6 females, **c**) and *Chd8*^+*/*+^ (+/+, *n* = 12; 6 males, 6 females, **c**) mice. *Chd8*^+*/*+^ animals are illustrated using filled circles, dotted lines with grey bars and *Chd8*^*neo/neo*^ animals with open triangles, solid lines and open bars. Apart from USVs (**c**), all other tests were conducted on young adults 6–14 weeks of age. **a** Duration, in seconds, spent in each chamber of the three-chamber sociability test. All mice spent a significantly higher proportion of time in the chamber with the age- and sex- matched stranger con-specific mouse compared to the other chambers. Mean ± SEM; *** < 0.001 (between-subjects ANOVA with student’s *t* test as post-hoc analysis). **b** Duration, in seconds, of social investigation over a three-minute period. Social investigation was defined as the combined total duration of head, body and anogenital sniffing of a conspecific mouse. Mean ± SEM (between-subjects ANOVA). **c** The mean number of ultrasonic vocalisations per minute on indicated postnatal days. Mean ± SEM (repeated-measures ANOVA). **d** Graph demonstrating the performance in the olfactory habituation/dishabituation test. Mean ± SEM (repeated-measures ANOVA). **e** The duration, in seconds, mice spent self-grooming during the 10-min self-grooming test. Mean ± SEM (between-subjects ANOVA). **f** The average number of marbles buried, out of a maximum of 12, within a 30-min period period. Mean ± SEM; * < 0.05, ** < 0.01 (repeated-measures ANOVA with student’s *t* test as post-hoc analysis). **g** Representative ethovision tracks of a *Chd8*^*neo/neo*^ (neo/neo) and *Chd8*^+*/*+^ (+ / +) animal plotting their movements during the 10-min open field task. **h** The percentage of time spent in the centre of the open field arena during the 10-min test. Mean ± SEM; **p < 0.01 (between-subjects ANOVA). **i** The total distance travelled in the outer part of the open field arena over a 10-min time-period. Mean ± SEM; *** < 0.001 (between-subjects ANOVA). **j** The total activity counts per 12 h period on running wheels in the homecage during 7 days of dark-phase recording. Mean ± SEM; *p < 0.05 (repeated-measures ANOVA). **k** The mean latency of mice to fall from the rotarod. Mean ± SEM (repeated-measures ANOVA)

To explore whether behavioural phenotypes may be exacerbated in the *Chd8*^*neo/neo*^ mild hypomorphs compared to *Chd8*^+/−^ mice, we assessed socio-communicative, repetitive, anxiety and motor behaviours. In total, three batches of *Chd8*^*neo/neo*^ mice were assessed. As no batch effects and no sexually dimorphic behaviours were observed, data from all three experiments and both sexes were combined for analysis. *Chd8* hypomorphs displayed normal social approach and investigation behaviours in the three-chamber (Fig. 2a) and reciprocal social investigation tests (Fig. 2b). Relative to wildtype littermates, hypomorph pups emitted normal numbers of ultrasonic vocalisations (USVs) on separation from the nest, suggesting no deficits in socio-communicative abilities (Fig. 2c). Furthermore, *Chd8*^*neo/neo*^ mice demonstrated a normal capacity for habituation and dishabituation to both social (sex-matched urine) and non-social (banana) odours (Fig. 2d). We did not observe any differences in repetitive self-grooming behaviour between hypomorphs and wildtypes (Fig. 2e). In the marble burying test, a task also designed to measure repetitive behaviours in mice, *Chd8*^*neo/neo*^ mice buried fewer marbles than controls (Fig. 2f). Interestingly, *Chd8* hypomorphs showed increased aversion towards the centre of the open field arena (Fig. 2g, h), suggesting that reduced *Chd8* expression may predispose to anxiety. In addition, these mice demonstrated decreased locomotor activity in the open field arena, even when controlling for their anxiety phenotype (Fig. 2i). This hypo-active phenotype was corroborated by homecage running wheel tests (Fig. 2j) and may account for the reduced marble burying observed in these mice. However, the hypo-activity did not appear to be due to motor defects, as these mice showed motor performance comparable to controls in the rotarod test (Fig. 2k).

In summary, *Chd8*^*neo/neo*^ mice showed no evidence of socio-communicative deficits or repetitive behaviours typically associated with ASD, suggesting that the reason for a lack of robust ASD-like behaviours in *Chd8*-deficient mouse models is unlikely to be an insufficient reduction in *Chd8* expression. The most robust, reproducible phenotype was locomotor hypoactivity, which was present in both our mild hypomorphic and *Chd8*^+/−^ mice [18], and also *Chd8*^+/−^ mice reported by Platt et al. [16]. Intriguingly, whereas our *Chd8*^+/−^ mice did not show clear anxiety phenotypes [18], the *Chd8* hypomorphs did, suggesting that this particular phenotype may be enhanced by a reduction in *Chd8* levels.

### Gradual reductions in CHD8 levels result in progressively more pronounced gene expression changes

To understand how sub-heterozygous *Chd8* levels affect gene expression in the embryonic neocortex, RNA-sequencing (RNA-seq) was performed from E12.5 neocortices. Data from heterozygous (*Chd8*^+/−^), mild (*Chd8*^*neo/neo*^) and severe (*Chd8*^*neo/−*^) hypomorphs, together with their respective wildtype littermate controls were included for analysis.

**Fig. 3:**
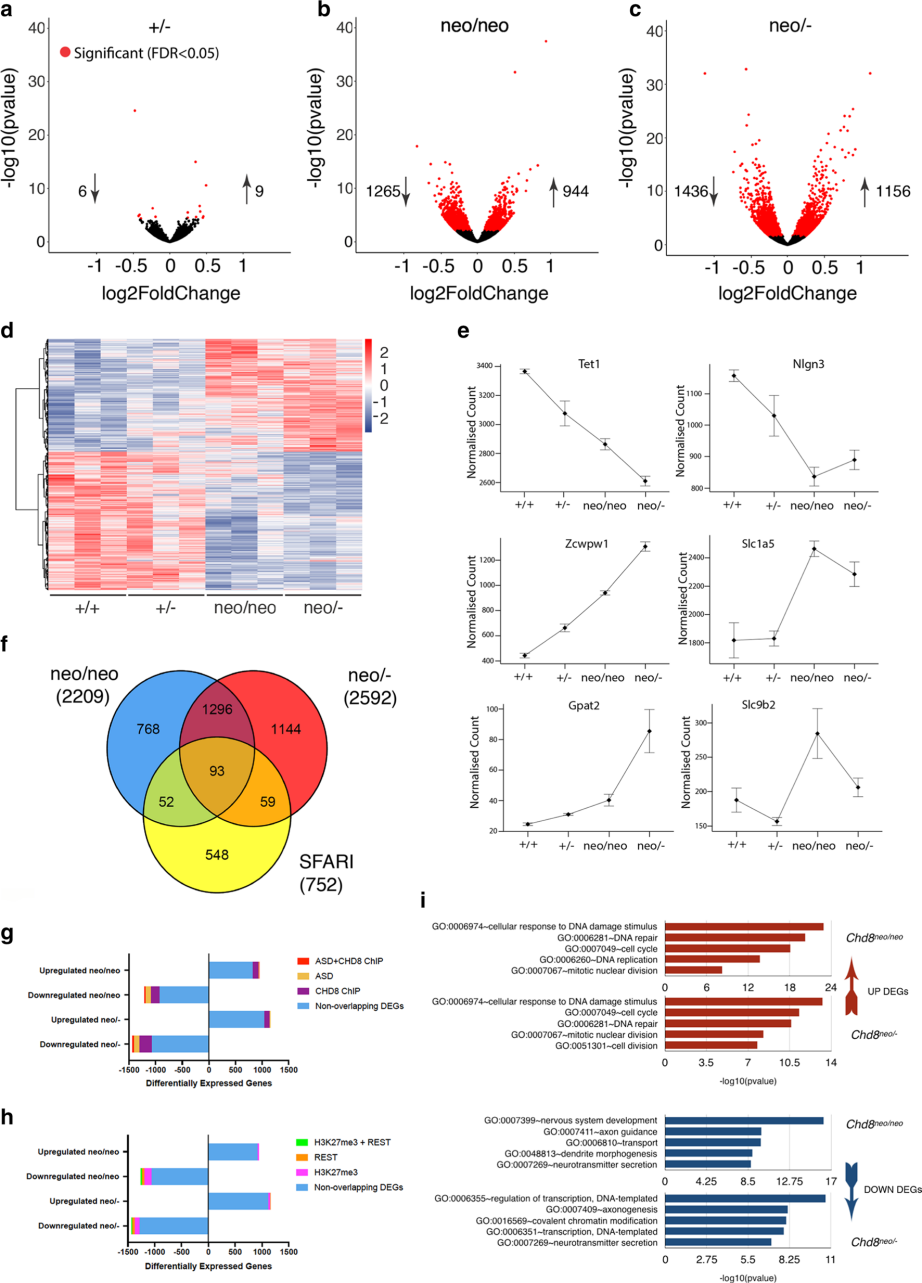
Gene expression changes in *Chd8-*deficient neocortices. **a** Volcano plot indicating all DEGs detected by DESeq2 in E12.5 *Chd8*^+/−^ embryonic cortices. Each point represents an individual gene, and all heterozygote DEGs (FDR < 0.05) are highlighted in red. *n* = 3 per condition. **b** Volcano plot showing all DESeq2 detected DEGs in E12.5 neo/neo cortex. All differentially expressed genes (FDR < 0.05) are highlighted in red. *n* = 3 per condition. **c** Volcano plot of all DESeq2 detected DEGs in E12.5 neo/− cortex. All differentially expressed genes (FDR < 0.05) are highlighted in red. *n* = 3 per condition. **d** Heatmap of genes differentially expressed in neo/neo and neo/− embryos, indicating transformed relative expression levels in +/+, +/−, neo/neo and neo/− embryos. **e** Mean normalised count of aligned RNA-seq reads for a selection of genes that were differentially expressed in the mild and severe hypomorphs. **f** Venn diagram showing extent of overlap between neo/neo and neo/− DEGs and ASD associated genes obtained from the SFARI Human Gene database (https://gene.sfari.org/autdb/HG_Home.do, accessed March 2018). **g** Breakdown of neo/neo and neo/− DEGs that are ASD associated, DEGs with known CHD8 binding sites in E17.5 mouse neocortex, human mid foetal cortex and NSCs; and DEGs that are both ASD associated and have known CHD8 binding sites. Downregulated genes are denoted by a negative number. **h** Breakdown of neo/neo and neo/− DEGs with H3K27me3 in neural progenitors, DEGs with known REST binding sites, and DEGs that have been shown to have both H3K27me3 in neural progenitors and REST binding sites. Downregulated genes are denoted by a negative number. **i** Gene Ontology (GO) analysis of up and down-regulated neo/neo and neo/− DEGs under the “Biological Processes” category. The five most significant hits are shown for each set. See also Additional file 4: Table S3

This analysis identified only 14 differentially expressed genes (DEGs, excluding *Chd8*, FDR < 0.05) in *Chd8*^+/−^ (Fig. 3a, Additional file 3: Table S2, Additional file 1: Fig. S3) and 2209 DEGs in *Chd8*^*neo/neo*^ neocortices (Fig. 3b, Additional file 3: Table S2, Additional file 1: Fig. S3), indicating that many genes only show significant transcriptional effects when CHD8 levels fall below 50%. In *Chd8*^*neo/−*^ embryos, 2592 DEGs (FDR < 0.05) were identified (Fig. 3c, Additional file 3: Table S2, Additional file 1: Fig. S3). The visualisation of differential gene expression in a heat map demonstrated the marked transcriptomic differences between heterozygotes and mild hypomorphs (Fig. 3d). DEGs could be divided into four groups based on their responses to reduced CHD8 levels: (1) genes that show a “linear” response to *Chd8* downregulation (e.g. *Tet1* and *Zcwpw1*, Fig. 3e, Additional file 1: Fig. S3C), (2) genes that are not significantly different in *Chd8*^+/−^ embryos but sharply up- or downregulated in *Chd8*^*neo/neo*^ embryos (e.g. *Nlgn3* and *Slc1a5*, Fig. 3e, see also Additional file 1: Fig. S3B), (3) genes that are only significantly dysregulated in *Chd8*^*neo/−*^ embryos (e.g. *Gpat2*), and (4) genes that exhibited non-linear responses (e.g. *Slc9b2,* Fig. 3e). The majority of DEGs (> 99%) fell within group 2, indicating that over 2000 genes showed a striking threshold response as CHD8 protein is reduced from heterozygotes to mild hypomorphs.

Comparing DEGs in *Chd8*^*neo/neo*^ and *Chd8*^*neo/−*^ samples identified 1389 genes common to both datasets (Fig. 3f), all of which were changed in the same direction. ASD-associated genes were highly enriched in the DEGs from both *Chd8*^*neo/neo*^ (145 genes, *p* = 1.32 × 10^*−*9^, OR = 1.83, Fisher’s exact test for count data, Fig. 3f, Additional file 3: Table S2) and *Chd8*^*neo/−*^ embryos (152 genes, *p* = 5.532 × 10^*−*7^, OR = 1.62, Fisher’s exact test for count data, Additional file 3: Table 2). Nearly half (46%) of these ASD-associated genes were common to *Chd8*^*neo/neo*^ and *Chd8*^*neo/−*^ mice (Fig. 3f). The majority (89% and 88%, respectively) of ASD-associated DEGs were downregulated (orange and red fractions in Fig. 3g, Additional file 3: Table S2), supporting the idea of CHD8 as an important positive regulator of neurodevelopmental, autism-associated genes [13, 14].

To determine if CHD8 functions as a positive or negative regulator of gene expression, we identified genes most likely directly regulated by CHD8, using ChIP-seq data from Cotney et al. [13]. From the Cotney dataset, a consensus set of gene promoters with CHD8 recruitment in E17.5 embryonic mouse brain, human midfetal cortex, and neural progenitor contexts was defined. This gene list was compared to the list of differentially expressed genes in *Chd8* hypomorphs. This analysis revealed that the majority of DEGs were not identified in the consensus CHD8 ChIP-seq dataset (blue and orange fractions in Fig. 3g), suggesting that most transcriptional changes are induced by indirect effects. This is consistent with previous observations and the prevailing view that CHD8 regulates the expression of many other chromatin and epigenetic modifiers [13, 14]. DEGs with CHD8 localisation to their promoters in the Cotney data (red and purple fractions in Fig. 3g), were present in both up- and down regulated gene sets, suggesting that CHD8 can function as both an activator and repressor of these genes during cortical development.

To identify potential transcriptional co-regulators and DNA-binding factors that may cooperate with CHD8 during embryonic cortical development, Gene Set Enrichment Analysis was performed using the “ENCODE and ChEA Consensus TFs from ChIP-X” database in Enrichr [36]. This analysis revealed an over-representation of E2F (E2F4, E2F6 and E2F1) targets in the upregulated genes (Additional file 1: Fig. S4, Additional file 3: Table S2). E2Fs compose a family of transcription factors with important roles in DNA replication, cell cycle progression and proliferation. CHD8 has been previously shown to be involved in E2F-dependent transcriptional activation, and is necessary for recruitment of the “activator” E2F transcription factors E2F1 and E2F3 to G1/S transition promoters [37]. Our findings suggest that CHD8 functions as a repressor of E2F-regulated genes in the developing cortex and implicate increased progenitor proliferation as a potential mechanism for the brain hyperplasia in these mice.

For downregulated genes, an over-representation of targets of REST (RE1-Silencing Transcription factor) and the Polycomb component Suz12 was seen (Additional file 1: Fig. S4, Additional file 3: Table S2). As Suz12 is essential for the activity and stability of the PRC2 complex, we asked if any of the DEGs are marked by the PRC2-repressive modification H3K27me3 in normal neural progenitor cells [38]. The majority of DEGs that were marked by H3K27me3 in neural progenitors were downregulated in hypomorphic mice (pink fractions in Fig. 3h). Thus, some genes positively regulated by CHD8 are Polycomb targets in neural progenitors. Although further mechanistic studies will be required to test this, these findings are consistent with CHD8 functioning as an antagonist of Polycomb repression as expected of a Trithorax family member. REST is a master regulator of neurodevelopment, has been shown to directly interact with CHD8, and is abnormally activated in *Chd8* haploinsufficient mouse brain [15]. Overlapping DEGs with REST ChIP-seq data [39], we found REST target genes predominantly amongst downregulated genes (orange and green fractions in Fig. 3h), supporting the notion that aberrant REST activation in *Chd8*-deficient embryonic brain may contribute to gene repression. Furthermore, 40% of the downregulated REST target genes are also marked by H3K27me3 in neural progenitor cells (green fractions in Fig. 3h), implying roles for both REST and Polycomb in the repression of these genes in *Chd8* hypomorphs.

To provide further insights into the biological processes affected by these gene expression changes, gene ontology analyses were performed. These identified a significant enrichment of cell cycle, DNA replication and repair genes in the upregulated genes in hypomorphs (Fig. 3i, Additional file 4: Table S3). Neurodevelopmental gene categories were enriched in the downregulated gene sets (Fig. 3i, Additional file 4: Table S3).

### *Chd8* deficiency increases proliferation of cortical progenitors outside of the ventricular zone

To explore whether the increased expression of cell cycle and DNA replication genes in hypomorphs is indeed associated with increased progenitor proliferation, we immunolabelled progenitors in the G2/M phase of mitosis in coronal brain sections with an antibody against phosphohistone-3B (PH3B). No difference in the number of mitotic progenitors was observed in E12.5 *Chd8*^+/−^ or *Chd8*^*neo/neo*^ neocortices, compared to wildtype littermates (Additional file 1: Fig. S5A–C). To determine if differences may arise later, we performed the same experiment at E14.5 (Additional file 1: Fig. S5D, E). Again, no significant differences were seen in the numbers of proliferating ventricular progenitors (Additional file 1: Fig. S5F). Intriguingly, a significant increase in the proliferation of non-ventricular (or basal) progenitors was observed in *Chd8*^*neo/neo*^ neocortices (Additional file 1: Fig. S5G). No significant differences were observed in *Chd8*^+/−^or *Chd8*^*neo/−*^ embryos Additional file 1: Fig. S5F, G), suggesting that the abnormal expansion of basal cortical progenitors only occurred within a restricted window of reduced CHD8 expression.

**Fig. 4:**
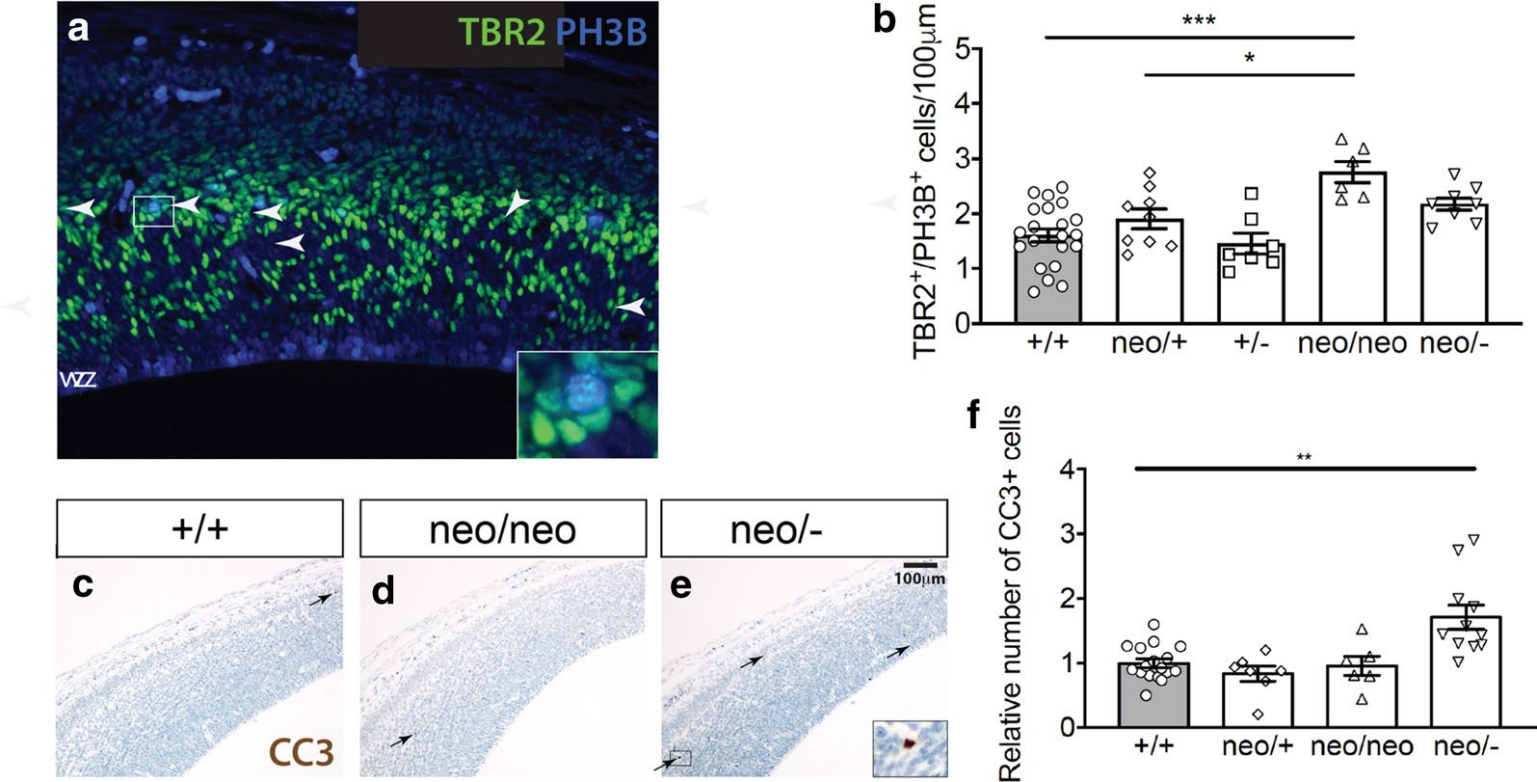
Increased proliferation of basal neural progenitors in *Chd8*^*neo/neo*^ embryos. **a** Immunohistochemistry to detect PH3B+  (blue) and TBR2+ (green) nuclei in coronal sections through the telencephalon of E14.5 embryos. White arrowheads indicate PH3B+/TBR2+nuclei. White box is shown as zoomed inset containing a double positive cell. **b** Quantification of TBR2+/PH3B+cells per 100 µm of neocortex in E14.5 embryos (+/+, *n* = 20; neo/+, *n* = 9; +/−, *n* = 7; neo/neo, *n* = 6; neo/−, *n* = 8; Mean ± SEM, *p < 0.05, ***p < 0.001, ANOVA followed by Tukey’s multiple comparisons test). **c**–**e** Cleaved caspase 3 (CC3) immunostaining of dorsal neocortex of E14.5 embryos. Black arrows indicate CC3+ cells and black box in E highlights area shown in zoomed inset containing a CC3+ cell. Scale bar = 100 μm. **f** Relative quantification of CC3+ cells in neocortex of E14.5 *Chd8* mutant embryos, normalised to respective wildtype littermates (+/+, *n* = 17; neo/ + , *n* = 7; neo/neo, *n* = 6; neo/−, *n* = 11; Mean ± SEM **p < 0.01). Embryos used in these experiments were from 8 different litters

To determine the identity of the non-ventricular progenitors that proliferated more in mild hypomorphs, we stained serial sections with antibodies to TBR2, a marker for intermediate progenitors, and PH3B (Fig. 4a). Counting these proliferating TBR2+ progenitors revealed a significant increase in the proliferation of these cells in *Chd8*^*neo/neo*^ embryos, compared to controls (Fig. 4b). No significant changes in the number of proliferating TBR2+ progenitors was observed in other genotypes (Fig. 4b). Taken together, these experiments showed that TBR2+ intermediate progenitors accounted for the increased non-ventricular proliferation observed in *Chd8*^*neo/neo*^ embryos.

Our data suggests that TBR2+ intermediate progenitors might be particularly sensitive to reduced *Chd8* expression. To find if *Chd8* deficiency directly impacts gene regulation in these cells, we sorted TBR2+ and PAX6+ progenitors from E14.5 *Chd8*^+*/*+^, *Chd8*^+/−^ and *Chd8*^*neo/neo*^ neocortices by FACS and compared gene expression by qRT-PCR. We selected two genes identified in our RNA-seq analysis that are upregulated in *Chd8*^*neo/neo*^ mice, *Pim1,* a gene functionally linked to progenitor cell proliferation [40] and *Axin2*, a direct transcriptional target and feedback antagonists of WNT-β-catenin signalling [41]. Both *Pim1* and *Axin2* were significantly upregulated in *Chd8*-deficient TBR2+, but not PAX6+ progenitors, with *Axin2* showing a clear *Chd8* dosage-sensitive effect (Additional file 1: Fig. S6B, C). Together, these findings show that *Chd8* deficiency impacts the expression of specific genes linked to neural progenitor proliferation and fate specifically in TBR2+ intermediate progenitors. The gene expression changes in *Chd8*^+/−^ embryos may not be sufficient to increase the proliferation of these progenitors, or may have consequences at later stages of development.

Next, we sought to identify the potential causes of cortical hypoplasia in severe hypomorphs (Additional file 1: Fig. S2E, G). We did not detect any reduction in cortical progenitor proliferation at E14.5 in these mutants (Fig. 4b). Comparing gene ontology analyses between mild and severe hypopmorphs (Additional file 1: Figs. S7 and S8; Additional file 4: Table S3), revealed a slight increase in the number of p53-regulated genes and ribosomal genes like *Rpl26* that can augment p53 mRNA translation [42] in severe hypomorphs, raising the possibility that progenitors may be more prone to apoptosis in these mice. Indeed, cleaved caspase 3 (CC3) immunostaining revealed a small increase in the numbers of apoptotic cells in *Chd8*^*neo/−*^ embryos compared to *Chd8*^*neo/neo*^ and control embryos (Fig. 4c–f), suggesting that increased cell death may be responsible for the brain hypoplasia in the severe hypomorphs. Thus, reducing CHD8 levels below 15% of wildtype, appeared to reveal another threshold below which p53-regulated genes become derepressed with an apparent impact on cell survival.

### CHD8 expression is essential for repression of p53 target genes in early embryonic neocortex

**Fig. 5:**
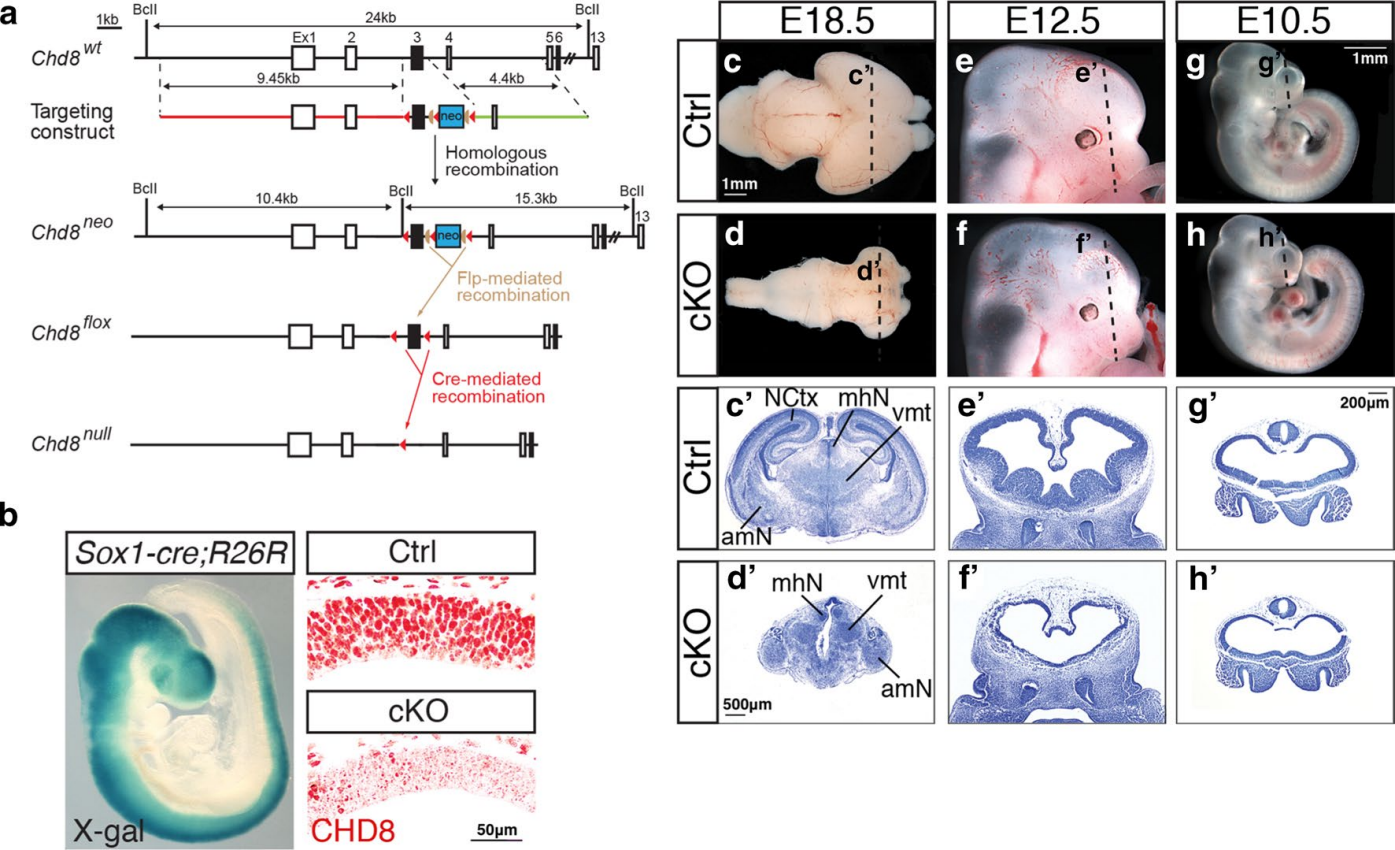
Conditional deletion of *Chd8* from the embryonic neuroepithelium results in severe hypoplasia of the telencephalon and neocortex. **a** Schematic representation of the wildtype (wt) mouse *Chd8* gene (*Chd8*^*wt*^), targeting construct for homologous recombination in embryonic stem cells, the *Chd8* targeted allele (*Chd8*^*neo*^), the *Chd8* conditional allele after Flp-mediated excision of the neomycin resistance cassette (*Chd8*^*flox*^) and the *Chd8*^*null*^ allele produced by Cre-mediated deletion of exon 3. Boxes represent exons, with exon 1 (Ex1) to 6 and 13 shown and exon 3 filled in black. The blue box represents a neomycin resistance cassette (neo), red triangles represent *loxP* sites and tan triangles *frt* sites. The long 9.45 kb (5′) homology arm is indicated in red and the short 4.4 kb (3′) homology arm in green in the targeting construct. **b** X-gal staining of a *Sox1-Cre; R26R* embryo at E9.5 (left); and immunostaining for CHD8 protein on *Chd8*^*flox/flox*^ (Ctrl) and conditional knockout *Sox1-Cre; Chd8*^*flox/flox*^ (cKO) E10.5 neural tube (right). Scale bar = 50 μm. **c**, **d** Wholemount images of E18.5 brains of a representative Ctrl and cKO embryo, anterior is to the right. Scale bar = 1 mm. **e**, **f** Wholemount images of embryonic day 12.5 heads, anterior to the right. Scale bar = 1 mm. **g**, **h** Wholemount images of E10.5 embryos, anterior to the right. **c**′–**h**′) Cresyl violet-stained frontal sections through brains as indicated in **c**–**h** above. Scale bars = 500 μm (**c**′, **d**′) and 200 μm (**e**′–**h**′). The following subcortical structures are labelled in Ctrl (**c**′) and cKO (**d**′) at E18.5: NCtx: Neocortex, mhN: medial habenular nucleus, vmt: ventral medial thalamic nucleus, amN: amygdaloid nucleus. Images are representative of at least 3 independent samples

As the brain phenotype and effects on p53-regulated genes and apoptosis in severe hypomorphs were subtle, we decided to explore the consequences of complete CHD8 loss (0%), by conditional deletion of *Chd8* in early neural progenitors. *Sox1-cre*-mediated deletion (Fig. 5a, b, Additional file 1: Fig. S9) of *loxP*-flanked (flox) exon 3 results in an early frameshift and termination of translation at amino acid 419, which is predicted to produce a protein that lacks all functional domains and results in a *Chd8*-null allele as shown previously in *Chd8*^+/−^ mice [18].

The pan-neuronal, conditional deletion of *Chd8* by *Sox1-cre* (Fig. 5b, Additional file 1: Fig. S9) resulted in pronounced brain hypoplasia in homozygous conditional knockout (cKO) embryos, compared to controls (Ctrl, *Chd8*^*flox/flox*^ (*Chd8*^*f/f*^)) (Fig. 5c, d). Neocortical hypoplasia, with the maintenance of some subcortical brain structures was evident upon histological analysis of E18.5 embryos (Fig. 5c′, d′). To identify the origin of these defects, cKO embryos were examined at earlier stages of development. Telencephalic hypoplasia with markedly thinner neuroepithelium was evident in E12.5 cKO embryos when compared to controls (Fig. 5e, f′). Examination of E10.5 cKO embryos showed telencephalic vesicles of near-normal size with neuroepithelia that were slightly thinner than controls (Fig. 5g–h′), suggesting that CHD8 is essential for expansion of the pallium from early embryonic development.

**Fig. 6:**
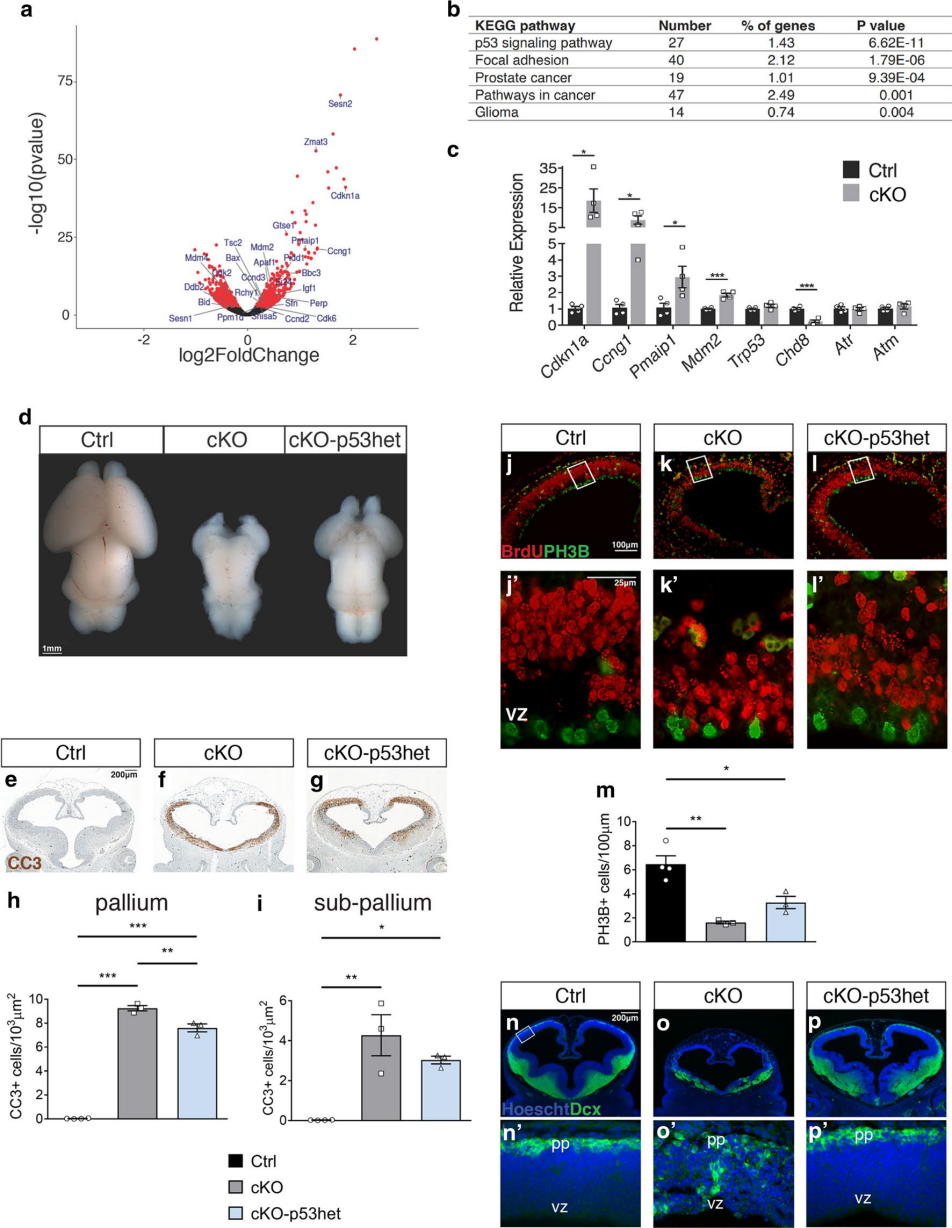
Repression of p53 target genes by CHD8 is necessary for normal brain growth. **a** Volcano plot of RNA-seq data illustrating in red genes that are differentially expressed (FDR < 0.05) in E10.5 cKO telencephalon, with p53 pathway genes labelled. **b** Pathway enrichment analysis of differentially expressed genes, with the top KEGG pathway terms shown [43]. See also Additional file 5: Table S4. **c** qRT-PCR validation of a selection of p53 pathway genes identified by RNA-seq (*n* = 4 for each condition. Mean ± SEM; *p < 0.05, ***p < 0.001, student’s *t* test). **d** Wholemount brains from E18.5 Ctrl, cKO and cKO-p53het mice are shown, anterior to the top. Data are representative of 6 embryos per genotype. Scale bar = 1 mm. **e**–**g** Cleaved caspase 3 (CC3) immunohistochemistry (brown) on frontal sections through the telencephalon of E12.5 embryos. Scale bar = 200 μm. **h** Quantification of CC3+ cells/μm^2^ of the pallium in embryos of each genotype (Ctrl, *n* = 4; cKO, *n* = 3; cKO-p53het, *n* = 3; Mean ± SEM; *p < 0.05, **p < 0.01, ***p < 0.001, ANOVA followed by Tukey’s multiple comparisons test). **i** Quantification of CC3+ cells/μm^2^ of the subpallium (medial ganglionic eminence) in embryos of each genotype (Ctrl, *n* = 4; cKO, *n* = 3; cKO-p53het, *n* = 3; Mean ± SEM; *p < 0.05, **p < 0.01, ANOVA followed by Tukey’s multiple comparisons test). **j**–**l** Immunohistochemistry to detect BrdU + (red) and phospho-histone 3B (PH3B)+ nuclei in frontal sections through the telencephalon of E12.5 embryos. Scale bar = 100 μm. **j**′–**l**′ Magnified images of the boxed neocortical regions in **j**–**l**, with the ventricular zone (vz) at the bottom and pial surface at the top. Scale bar = 25 μm. **m** Quantification of PH3B+ cells/μm of neocortical ventricular surface (Ctrl, *n* = 4; cKO, *n* = 3; cKO-p53het, *n* = 3; Mean ± SEM; *p < 0.05, **p < 0.01, ANOVA followed by Tukey’s multiple comparisons test). **n**, **p** Immunostaining of frontal E12.5 sections for DCX (green) to label differentiating neurons, with nuclei counterstained with Hoechst 33,342. Scale bar = 200 μm. **n**′, **p**′ Magnified images of **n**, **p**, with the ventricular zone (vz) at the bottom and pial surface (pp) at the top. Scale bar = 25 μm

To identify the potential causes of this striking phenotype, we performed RNA-seq at the onset of the phenotype. RNA-seq analysis identified 2032 DEGs in E10.5 cKO telencephalic vesicles compared to controls (Fig. 6a, Additional file 5: Table S4). KEGG pathway mapping of all dysregulated DEGs identified the p53 pathway as the most significantly affected (Fig. 6b, Additional file 1: Fig. S10). Interestingly, GO analysis identified cell cycle as the most dysregulated bioprocess (Additional file 5: Table S4), with a slight majority of genes within this category downregulated (60 out of 111). Quantitative RT-PCR (qRT-PCR) confirmed significant upregulation of multiple p53-regulated genes (Fig. 6c). Furthermore, genes normally upstream of p53, *Atr* and *Atm,* and *Trp53* (the gene encoding p53 itself) were not affected by *Chd8* deletion (Fig. 6c), consistent with a role for CHD8 in directly repressing p53 target genes [10].

To ask if increased p53 pathway activity was responsible for the cKO phenotype, we reduced p53 gene expression in the neuronal lineage to test if this can reduce the severity or incidence of phenotypic abnormalities. Neocortical hypoplasia was partially rescued in *Sox1-Cre; Chd8*^*f/f*^*; Trp53*^*f/*+^ (conditional knockout p53 heterozygous, cKO-p53het) embryos (Fig. 6d), providing strong genetic evidence that this phenotype was caused by elevated p53 signaling. A substantial increase in apoptosis was observed in the cKO embryos (Fig. 6e, f, h, i). The number of apoptotic cells was significantly reduced in the pallium of cKO-p53het embryos, compared to cKO embryos (Fig. 6f–i). We also noted the presence of certain cell cycle inhibitors amongst genes upregulated in the cKO (e.g. p21/CDKN1A) (Fig. 6c). Therefore, we investigated neural progenitor proliferation. Quantification of PH3B+ cells in the ventricular zone of the neocortex confirmed a strong reduction in cell proliferation in cKO embryos (Fig. 6j, j′, k, k′, m). Cell proliferation was slightly increased in cKO-p53het embryos compared to cKO embryos (Fig. 6l, l′, m), consistent with only a partial rescue of neocortical size in these animals (Fig. 6d). The visualisation of proliferating BrdU+ cells in the same sections appear to support this conclusion (Fig. 6j–l′).

To determine if neuronal differentiation was affected in these mutants, we immunostained for Doublecortin (DCX). DCX+ cells were present in the preplate (pp) at the pial surface in all control embryos (*n* = 3/3; Fig. 6n, n′). Ectopic clusters of DCX+ cells were visible throughout the neural tube of all cKO embryos (*n* = 4/4), including the ventricular zone (Fig. 6o, o′), suggesting a precocious and disorganised differentiation of progenitors compared to controls (*p* = 0.029, Fisher’s exact test). DCX+ cell positioning was normalised in all cKO-p53het embryos (*n* = 4/4; Fig. 6p, p′) indicating a significant rescue of this phenotype compared to cKO embryos (*p* = 0.029, Fisher’s exact test).

Taken together, these data identify CHD8 as an essential repressor of p53 pathway activation during neocortical development. CHD8 loss leads to increased apoptosis, reduced neural progenitor proliferation and precocious cell differentiation during early embryonic development, resulting in severe neocortical hypoplasia by the end of gestation.

## Discussion

**Fig. 7:**
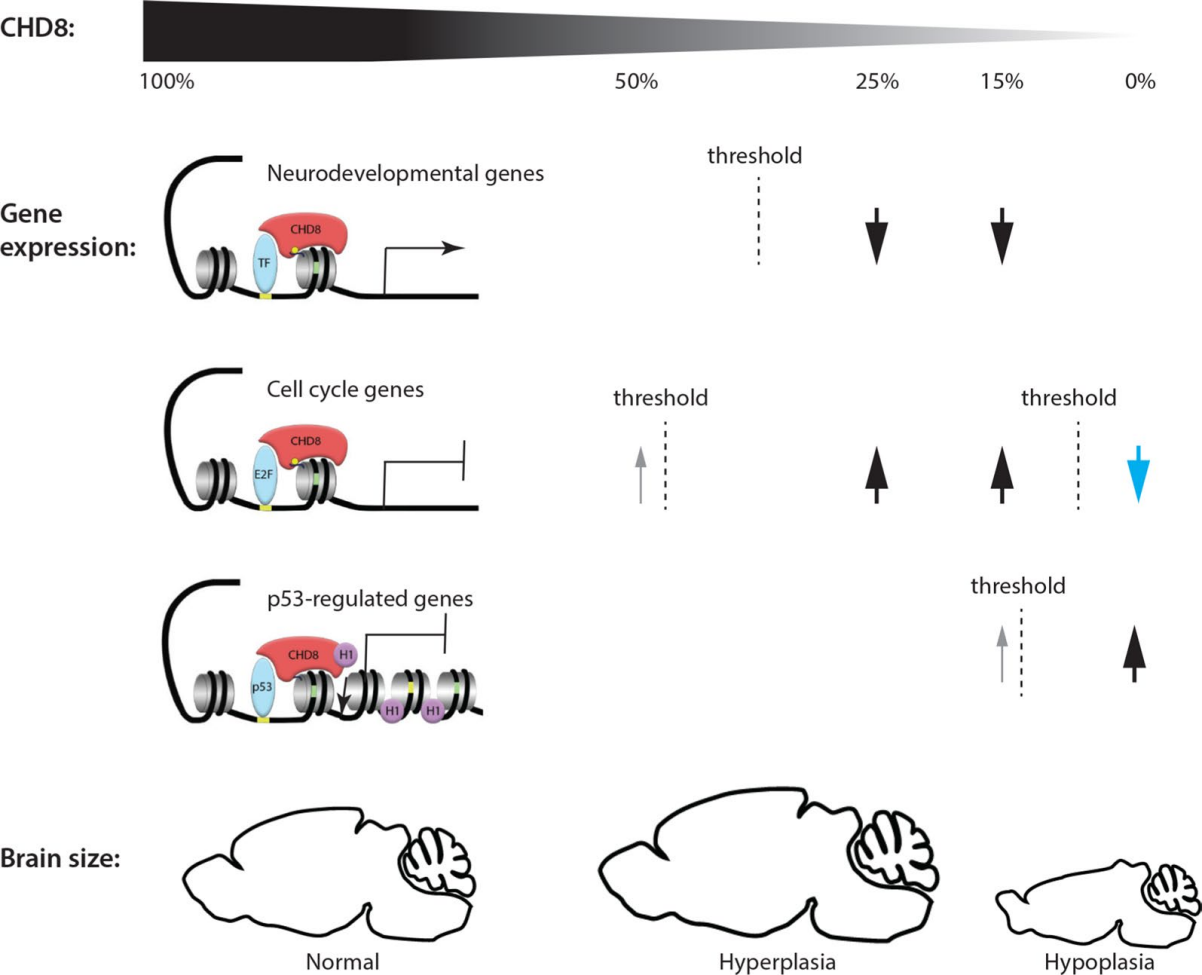
The non-monotonic relationship between CHD8 protein levels, gene expression and brain size. The effects of gradual reductions in CHD8 protein levels to ~ 50% (heterozygous), ~ 35% (mild hypomorph), ~ 10% (severe hypomorph) and 0% (conditional knockout) on the transcription of neurodevelopmental, cell cycle and p53-regulated genes and brain size are depicted. CHD8 appears to function primarily as a positive regulator of neurodevelopmental genes via recruitment to H3K4me3-modified (yellow ball) histones (gray spool), presumably via enabling the recruitment of key transcription factors (TF). A sharp reduction in the expression of many of these genes (arrow) is only observed in E12.5 neocortex when CHD8 levels are reduced to below a threshold less than haploinsufficient levels. CHD8 appears to repress E2F-regulated cell cycle genes in this context, with significant induction only becoming evident at sub-haploinsufficient levels, although low expression increases (grey arrow) likely drives subtle increases in proliferation in the heterozygous state. Cell cycle genes are dysregulated in the opposite direction in the cKO, suggestive of non-monotonic effects (blue arrow). CHD8 can interact with p53 and histone 1 (H1), leading to stable heterochromatin formation and repression of p53 target genes. A few p53-regulated genes become activated in hypomorphic mice (grey arrow), but the majority remains fully repressed with de-repression only becoming evident upon complete CHD8 loss. Note the different CHD8 thresholds for different groups of genes (broken lines) and the non-monotonic effects on gene expression and over-all brain size

Human genetic studies have identified heterozygous, likely gene disrupting mutations in *CHD8* as a possible cause of ASD and macrocephaly. *Chd8* heterozygous mice have been generated by several groups, but these mice were found to exhibit relatively subtle brain overgrowth [15–17, 19]. Observations of relatively small transcriptional changes in the mid-gestation *Chd8*^+/−^ mouse brain appeared at odds with the many genes dysregulated upon *Chd8* knockdown in progenitor cell lines and after in utero electroporation [13, 14, 20]. Together, these studies led us to explore whether different sensitivities to reduced CHD8 dosage might account for some of these inconsistencies. A comparison of brain size, gene expression and neural progenitor fate in a mouse *Chd8* allelic series yielded several key findings: (1) A small additional reduction in *Chd8* expression in mild hypomorphs compromised the capacity of neural progenitor cells to maintain stable expression of over 2000 genes in the mid-embryonic neocortex, which included over 140 ASD-associated genes; (2) CHD8 has a key role in limiting the expansion of TBR2+ intermediate progenitors, a population particularly important for human cortical development; (3) A gradual reduction in CHD8 expression can have non-linear effects on gene expression and brain growth (Fig. 7). In addition to the precipitous gene expression changes in mild hypomorphs, we observed brain hyperplasia in *Chd8*^+/−^ and *Chd8*^*neo/neo*^ mice, but brain hypoplasia in severe hypomorphs and conditional knockouts (Fig. 7). Together, these findings indicate that CHD8 levels need to be tightly regulated during development and that the interpretation of experimental manipulations that involve *Chd8* knock-down should consider these non-linear, threshold effects.

### CHD8 as a phenotypic capacitor

It has been posited previously that ASD-associated chromatin remodelling factors may act as phenotypic capacitors, buffering against perturbations to normal development in order to maintain stable phenotypes [44]. Heterozygosity for a capacitor is predicted to result in a loss of robustness, such that the cells are more susceptible to additional genetic and non-genetic risk factors. Our findings that over 2200 genes, many of which are ASD risk factors, became dysregulated by a small additional decrease in CHD8 dosage below 50%, supports the idea that some neurodevelopmental genes and processes may be close to a critical threshold in the *Chd8* heterozygous neocortex and therefore easily perturbed by small additional changes. It will be important to determine if specific ASD-associated *CHD8* mutations in humans reduce CHD8 function by more than 50% by dominant negative mechanisms, as our findings would predict these mutations to be significantly more pathogenic than purely haploinsufficient mutations. It is also important to consider that the C57BL/6 genetic background has been used for all *Chd8* heterozygous mouse studies so far. This background may be protective and more robust in the context of *Chd8* haploinsufficiency, such that different, or more severe phenotypes may emerge on different genetic backgrounds. We report a significant effect of sub-haploinsufficient levels of *Chd8* expression on the proliferation of intermediate progenitors in the E14.5 embryonic cortex. It will be of interest to determine if other cell types relevant to CHD8 function in the brain, like oligodendrocytes [45], exhibit similar *Chd8* gene dosage sensitive responses.

### CHD8 regulates the proliferation of non-ventricular cortical progenitors

Our findings raise the possibility that brain development in human and mouse differ in sensitivity to a similar reduction in CHD8 function, such that *CHD8* heterozygosity may cause more pronounced changes to brain growth and transcriptional regulation in the developing human brain. In this regard, fundamental differences in mouse and human brain development may result in *CHD8* haploinsufficiency having more pronounced effects on human brain development. Comparative studies of gyrencephalic and lissencephalic animals have identified important differences in the capacity of non-ventricular progenitors to expand and subsequently contribute to cortical expansion. This population of progenitors consists of outer radial glia cells (oRGs), and TBR2-expressing intermediate progenitor cells (IPs) [46]. In humans, oRGs are located in an expanded outer-subventricular zone (oSVZ) and are capable of asymmetric divisions that generate an oRG daughter cell, which maintains the pool of non-ventricular progenitors, and an IP daughter cell that can undergo transit-amplifying divisions to expand and generate neuronal progeny [47]. By contrast, mouse oRGs primarily undergo self-renewing, neurogenic divisions and populate a non-ventricular region lacking the distinct, expanded cytoarchitecture of the oSVZ typically seen in gyrencephalic species [48]. Furthermore, mouse IPs likely possess a more limited capacity for self-renewal, as a majority of mouse IP divisions appear to generate two neuronal daughter cells [49, 50]. Therefore, if CHD8 has an especially crucial role in regulating the expansion of TBR2+ progenitors also in the human brain, then it is possible that CHD8 deficiency in these cells could result in more pronounced phenotypes with regard to cortical over-growth and circuit disruption in humans. The lack of exacerbated ASD-linked behavioural phenotypes in *Chd8*^*neo/neo*^ mice is consistent with this notion.

Interestingly, we also note that Bernier et al. previously identified an enrichment for CHD8 expression in areas outside the ventricular zone in human mid-fetal cortex [6], further supporting the idea that CHD8 may have an important role in regulating expansion of these cells. An analysis of neural progenitor subsets in *CHD8*-deficient human brain organoids should be a viable way to test this hypothesis.

### CHD8 is an essential repressor of p53 in neural progenitors

One of the most striking non-monotonic effects of *Chd8* efficiency reported here is brain hypoplasia in pan-neuronal *Chd8* cKO mice, partly as a result of de-repression of the p53 pathway in early neuroepithelial cells. This discovery identifies CHD8 as a critical repressor of p53 target gene activation in neural progenitors. Our findings suggest that very low levels of CHD8 are sufficient to repress p53 target genes and maintain neural progenitor self-renewal. One could speculate that the CHD8-dependent recruitment of histone H1 to p53 target genes to initiate a cooperative process of chromatin compaction [10], may require lower levels of CHD8 than another process that is dependent upon the constitutive recruitment of RNA polymerase [51] or other co-activating factors by CHD8 (Fig. 7). Our gene expression and apoptosis data suggest that CHD8 protein levels in *Chd8*^*neo/−*^ embryos were close to this critical threshold. Interestingly, Cotney et al. also reported p53 signaling as one the most dysregulated pathways upon CHD8 knock-down in human neural stem cells [13]. However, other studies in human cell lines have not demonstrated the same changes, including an in vitro knock-down of CHD8 to 20–25% of control levels in human SK-N-SH neural progenitor cells [52]. Intriguingly, CHD8, and its family member CHD7, also maintains the survival of oligodendrocyte precursors by inhibiting the p53 pathway, although this appears to be primarily mediated via direct repression of the p53 gene [53]. Together, these findings suggest that transcriptional responses to reduced CHD8 levels are highly context-dependent and may help shed light on reports that *Chd8* knock-down in utero led to reduced proliferation and enhanced differentiation of neural progenitors [20].

### Behavioural phenotypes in *Chd8*-deficient mice

A number of *Chd8*^+/−^ mouse models have been reported. Given the strong association between CHD8 mutations and ASD in humans, one might have expected these mice to exhibit robust, ASD-like behaviours. However, that is not the case, a finding not that surprising if one takes into account the pronounced differences in brain size and behaviour between these species. Nevertheless, we tested whether mice with more pronounced brain and gene expression phenotypes exhibit more pronounced behavioural phenotypes. With the possible exception of anxiety, the behavioural phenotypes of *Chd8*^+/−^ and hypomorphic mice were remarkably similar, including robust, reproducible hypo-activity [18].

## Limitations

As with all other *Chd8* mouse studies so far, our models were on a C57BL/6 genetic background. Different, or more severe phenotypes may emerge on different genetic backgrounds. Given the significant postnatal lethality of *Chd8* hypomorphic mice, we cannot exclude the possibility that the reason for not observing a particularly prominent exacerbation of behavioural phenotypes compared to heterozygous mice, may be that only mildly affected, surviving mice could be behaviourally phenotyped. Furthermore, unlike *Chd8*^+/−^ mice, all *Chd8*^*neo/neo*^ mice were born to *Chd8*^*neo/*+^ mothers, and the possibility that differences in maternal care could account for subtle behavioural differences cannot be ruled out. Recent work have reported sexually dimorphic effects in another *Chd8* model [19]. We did not observe significant behavioural differences between males and females in our studies, and the structural, molecular and developmental experiments reported here were performed with mixed sex samples. We therefore cannot rule out sex-specific effects in gene expression and perhaps even developmental phenotypes. Our findings identify an important role for the autism-associated factor CHD8 in controlling the proliferation of intermediate progenitors in the mouse neocortex. Our analysis of TBR2+ intermediate progenitors show a robust increase in the proliferation of these cells in *Chd8* hypomorphs, but not *Chd8*^+/−^ mice (Fig. 4), despite the latter showing significant alterations in gene expression (Additional file 1: Fig. S6). We cannot at this stage rule out the possibility that the dynamics of neural progenitor proliferation and differentiation differ in heterozygous and hypomorphic mice, and that intermediate progenitors over-proliferate also in *Chd8*^+/−^ mice, but at a different developmental stage. A comprehensive, timed analysis of TBR2+ progenitor fate will be required to test this possibility. We propose that CHD8 also regulates intermediate progenitor proliferation in human brain development, as indeed suggested by CHD8 expression studies [6], but studies on human cells are required to confirm this. Finally, we show that CHD8 represses p53-regulated genes and that p53 pathway hyperactivation in neuroepithelial cells that lack CHD8 is at least in part responsible for cellular phenotypes that can lead to cortical hypoplasia. These findings do not exclude the contribution of other pathways and cellular mechanisms to the hypoplasia phenotype.

## Conclusion

In conclusion, our analysis of an allelic series of *Chd8*-deficient mice has identified clear non-monotonic effects on gene expression and brain growth (Fig. 7). Recognition of the differing sensitivities of important cellular processes to CHD8 dosage and how small differences in CHD8 levels may lead to disproportionally large differences in phenotype is an important step in understanding the context-specific transcriptional roles of CHD8 in brain development.

## Supplementary information

**Additional file 1:** Supplementary information.

**Additional file 2: Table S1.** Raw MRI volumetric data, accompanies Fig. 1d, e.

**Additional file 3: Table S2.** RNA-seq data from E12.5 neocortices, accompanies Fig. 3.

**Additional file 4: Table S3.** Gene ontology and pathway analyses of E12.5 RNA-seq data, accompanies Fig. 3I.

**Additional file 5: Table S4.** RNA-seq and gene ontology analysis of E10.5 cKO telencephalic vesicles, accompanies Fig. 6a, b.

## Data Availability

The RNAseq raw data and read counts were deposited at the Gene Expression Omnibus (GEO) archive under the accession number GSE121381. All other materials will be made available upon reasonable request to the corresponding author.

## References

[1] O'Roak BJ, Vives L, Fu W, Egertson JD, Stanaway IB, Phelps IG (2012). Multiplex targeted sequencing identifies recurrently mutated genes in autism spectrum disorders. Science.

[2] Neale BM, Kou Y, Liu L, Ma'ayan A, Samocha KE, Sabo A (2012). Patterns and rates of exonic de novo mutations in autism spectrum disorders. Nature.

[3] Talkowski ME, Rosenfeld JA, Blumenthal I, Pillalamarri V, Chiang C, Heilbut A (2012). Sequencing chromosomal abnormalities reveals neurodevelopmental loci that confer risk across diagnostic boundaries. Cell.

[4] O'Roak BJ, Vives L, Girirajan S, Karakoc E, Krumm N, Coe BP (2012). Sporadic autism exomes reveal a highly interconnected protein network of de novo mutations. Nature.

[5] Iossifov I, O'Roak BJ, Sanders SJ, Ronemus M, Krumm N, Levy D (2014). The contribution of de novo coding mutations to autism spectrum disorder. Nature.

[6] Bernier R, Golzio C, Xiong B, Stessman HA, Coe BP, Penn O (2014). Disruptive CHD8 mutations define a subtype of autism early in development. Cell.

[7] Stessman HA, Xiong B, Coe BP, Wang T, Hoekzema K, Fenckova M (2017). Targeted sequencing identifies 91 neurodevelopmental-disorder risk genes with autism and developmental-disability biases. Nat Genet.

[8] Thompson BA, Tremblay V, Lin G, Bochar DA (2008). CHD8 is an ATP-dependent chromatin remodeling factor that regulates beta-catenin target genes. Mol Cell Biol.

[9] Sakamoto I, Kishida S, Fukui A, Kishida M, Yamamoto H, Hino S (2000). A novel beta-catenin-binding protein inhibits beta-catenin-dependent Tcf activation and axis formation. J Biol Chem.

[10] Nishiyama M, Oshikawa K, Tsukada Y, Nakagawa T, Iemura S, Natsume T (2009). CHD8 suppresses p53-mediated apoptosis through histone H1 recruitment during early embryogenesis. Nat Cell Biol.

[11] Nishiyama M, Nakayama K, Tsunematsu R, Tsukiyama T, Kikuchi A, Nakayama KI (2004). Early embryonic death in mice lacking the beta-catenin-binding protein Duplin. Mol Cell Biol.

[12] Nishiyama M, Skoultchi AI, Nakayama KI (2012). Histone H1 recruitment by CHD8 is essential for suppression of the Wnt-beta-catenin signaling pathway. Mol Cell Biol.

[13] Cotney J, Muhle RA, Sanders SJ, Liu L, Willsey AJ, Niu W (2015). The autism-associated chromatin modifier CHD8 regulates other autism risk genes during human neurodevelopment. Nat Commun.

[14] Sugathan A, Biagioli M, Golzio C, Erdin S, Blumenthal I, Manavalan P (2014). CHD8 regulates neurodevelopmental pathways associated with autism spectrum disorder in neural progenitors. Proc Natl Acad Sci USA.

[15] Katayama Y, Nishiyama M, Shoji H, Ohkawa Y, Kawamura A, Sato T (2016). CHD8 haploinsufficiency results in autistic-like phenotypes in mice. Nature.

[16] Platt RJ, Zhou Y, Slaymaker IM, Shetty AS, Weisbach NR, Kim JA (2017). Chd8 mutation leads to autistic-like behaviors and impaired striatal circuits. Cell Rep.

[17] Gompers AL, Su-Feher L, Ellegood J, Copping NA, Riyadh MA, Stradleigh TW (2017). Germline Chd8 haploinsufficiency alters brain development in mouse. Nat Neurosci.

[18] Suetterlin P, Hurley S, Mohan C, Riegman KLH, Pagani M, Caruso A (2018). Altered neocortical gene expression, brain overgrowth and functional over-connectivity in chd8 haploinsufficient mice. Cereb Cortex.

[19] Jung H, Park H, Choi Y, Kang H, Lee E, Kweon H (2018). Sexually dimorphic behavior, neuronal activity, and gene expression in Chd8-mutant mice. Nat Neurosci.

[20] Durak O, Gao F, Kaeser-Woo YJ, Rueda R, Martorell AJ, Nott A (2016). Chd8 mediates cortical neurogenesis via transcriptional regulation of cell cycle and Wnt signaling. Nat Neurosci.

[21] Hevner RF (2019). Intermediate progenitors and Tbr2 in cortical development. J Anat.

[22] Arnold SJ, Huang GJ, Cheung AF, Era T, Nishikawa S, Bikoff EK (2008). The T-box transcription factor Eomes/Tbr2 regulates neurogenesis in the cortical subventricular zone. Genes Dev.

[23] Marino S, Vooijs M, van Der Gulden H, Jonkers J, Berns A (2000). Induction of medulloblastomas in p53-null mutant mice by somatic inactivation of Rb in the external granular layer cells of the cerebellum. Genes Dev.

[24] Lewandoski M, Martin GR (1997). Cre-mediated chromosome loss in mice. Nat Genet.

[25] Truett GE, Heeger P, Mynatt RL, Truett AA, Walker JA, Warman ML (2000). Preparation of PCR-quality mouse genomic DNA with hot sodium hydroxide and tris (HotSHOT). Biotechniques.

[26] Genovese CR, Lazar NA, Nichols T (2002). Thresholding of statistical maps in functional neuroimaging using the false discovery rate. NeuroImage.

[27] Brown RZ (1953). Social behaviour, reproduction and population changes in the house mouse. Ecol Monogr.

[28] Krueger F. Trim Galore! 2012. Available from: http://www.bioinformatics.babraham.ac.uk/projects/trim_galore/.

[29] Liao Y, Smyth GK, Shi W (2014). featureCounts: an efficient general purpose program for assigning sequence reads to genomic features. Bioinformatics.

[30] Kim D, Pertea G, Trapnell C, Pimentel H, Kelley R, Salzberg SL (2013). TopHat2: accurate alignment of transcriptomes in the presence of insertions, deletions and gene fusions. Genome Biol.

[31] Love MI, Huber W, Anders S (2014). Moderated estimation of fold change and dispersion for RNA-seq data with DESeq2. Genome Biol.

[32] da Huang W, Sherman BT, Zheng X, Yang J, Imamichi T, Stephens R (2009). Extracting biological meaning from large gene lists with DAVID. Curr Protoc Bioinform.

[33] Meyers EN, Lewandoski M, Martin GR (1998). An Fgf8 mutant allelic series generated by Cre- and Flp-mediated recombination. Nat Genet.

[34] Murthy V, Tebaldi T, Yoshida T, Erdin S, Calzonetti T, Vijayvargia R (2019). Hypomorphic mutation of the mouse Huntington's disease gene orthologue. PLoS Genet.

[35] Kasah S, Oddy C, Basson MA (2018). Autism-linked CHD gene expression patterns during development predict multi-organ disease phenotypes. J Anat.

[36] Chen EY, Tan CM, Kou Y, Duan Q, Wang Z, Meirelles GV (2013). Enrichr: interactive and collaborative HTML5 gene list enrichment analysis tool. BMC Bioinform.

[37] Subtil-Rodriguez A, Vazquez-Chavez E, Ceballos-Chavez M, Rodriguez-Paredes M, Martin-Subero JI, Esteller M (2014). The chromatin remodeller CHD8 is required for E2F-dependent transcription activation of S-phase genes. Nucleic Acids Res.

[38] Mohn F, Weber M, Rebhan M, Roloff TC, Richter J, Stadler MB (2008). Lineage-specific polycomb targets and de novo DNA methylation define restriction and potential of neuronal progenitors. Mol Cell.

[39] Johnson R, Teh CH, Kunarso G, Wong KY, Srinivasan G, Cooper ML (2008). REST regulates distinct transcriptional networks in embryonic and neural stem cells. PLoS Biol.

[40] An N, Lin YW, Mahajan S, Kellner JN, Wang Y, Li Z (2013). Pim1 serine/threonine kinase regulates the number and functions of murine hematopoietic stem cells. Stem Cells.

[41] Jho EH, Zhang T, Domon C, Joo CK, Freund JN, Costantini F (2002). Wnt/beta-catenin/Tcf signaling induces the transcription of Axin2, a negative regulator of the signaling pathway. Mol Cell Biol.

[42] Takagi M, Absalon MJ, McLure KG, Kastan MB (2005). Regulation of p53 translation and induction after DNA damage by ribosomal protein L26 and nucleolin. Cell.

[43] Kanehisa M, Goto S (2000). KEGG: kyoto encyclopedia of genes and genomes. Nucleic Acids Res.

[44] Suliman R, Ben-David E, Shifman S (2014). Chromatin regulators, phenotypic robustness, and autism risk. Front Genet.

[45] Kawamura A, Katayama Y, Nishiyama M, Shoji H, Tokuoka K, Ueta Y (2020). Oligodendrocyte dysfunction due to Chd8 mutation gives rise to behavioral deficits in mice. Hum Mol Genet.

[46] Florio M, Huttner WB (2014). Neural progenitors, neurogenesis and the evolution of the neocortex. Development.

[47] Hansen DV, Lui JH, Parker PR, Kriegstein AR (2010). Neurogenic radial glia in the outer subventricular zone of human neocortex. Nature.

[48] Wang X, Tsai JW, LaMonica B, Kriegstein AR (2011). A new subtype of progenitor cell in the mouse embryonic neocortex. Nat Neurosci.

[49] Kowalczyk T, Pontious A, Englund C, Daza RA, Bedogni F, Hodge R (2009). Intermediate neuronal progenitors (basal progenitors) produce pyramidal-projection neurons for all layers of cerebral cortex. Cereb Cortex.

[50] Miyata T, Kawaguchi A, Saito K, Kawano M, Muto T, Ogawa M (2004). Asymmetric production of surface-dividing and non-surface-dividing cortical progenitor cells. Development.

[51] Rodriguez-Paredes M, Ceballos-Chavez M, Esteller M, Garcia-Dominguez M, Reyes JC (2009). The chromatin remodeling factor CHD8 interacts with elongating RNA polymerase II and controls expression of the cyclin E2 gene. Nucleic Acids Res.

[52] Wilkinson B, Grepo N, Thompson BL, Kim J, Wang K, Evgrafov OV (2015). The autism-associated gene chromodomain helicase DNA-binding protein 8 (CHD8) regulates noncoding RNAs and autism-related genes. Transl Psychiatry.

[53] Marie C, Clavairoly A, Frah M, Hmidan H, Yan J, Zhao C (2018). Oligodendrocyte precursor survival and differentiation requires chromatin remodeling by Chd7 and Chd8. Proc Natl Acad Sci U S A.

