# Supplementary material for: Distinct, dosage-sensitive requirements for the autism-associated factor CHD8 during cortical development
Source: Mol Autism. 2021 Feb 24;12:16. doi: 10.1186/s13229-020-00409-3 (PMC7905672; doi:10.1186/s13229-020-00409-3)
Supplement: Supplementary file 1 — Additional file 1: Supplementary information. [file 13229_2020_409_MOESM1_ESM.docx]

**Additional file 1: Supplementary information**

**
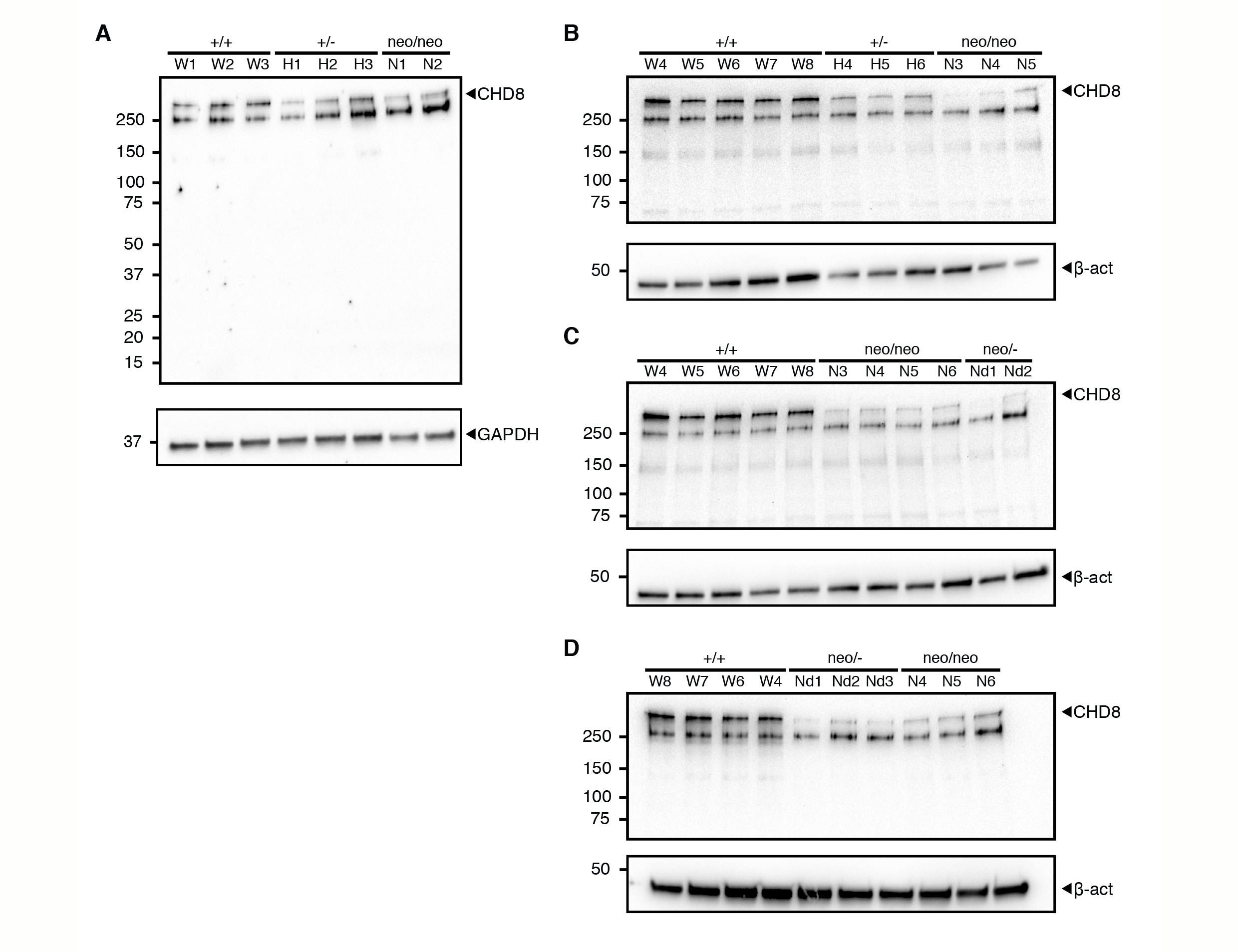
**

**Additional file 1: Figure S1. CHD8 expression is reduced in *Chd8^+/-^*, *Chd8^neo/neo^ and Chd8^neo/-^*  embryo telencephalic vesicles at E12.5**

Whole cell lysate from telencephalic vesicles of indicated genotypes were subjected to western blot analysis using an anti-CHD8 antibody targeting the N-terminal portion of the protein, utilising either GAPDH (A) or β-actin as a loading control (B-D). Arrows indicate bands corresponding to target protein. The band just below is a non-specific band. Labels above individual wells indicate biological replicates used.


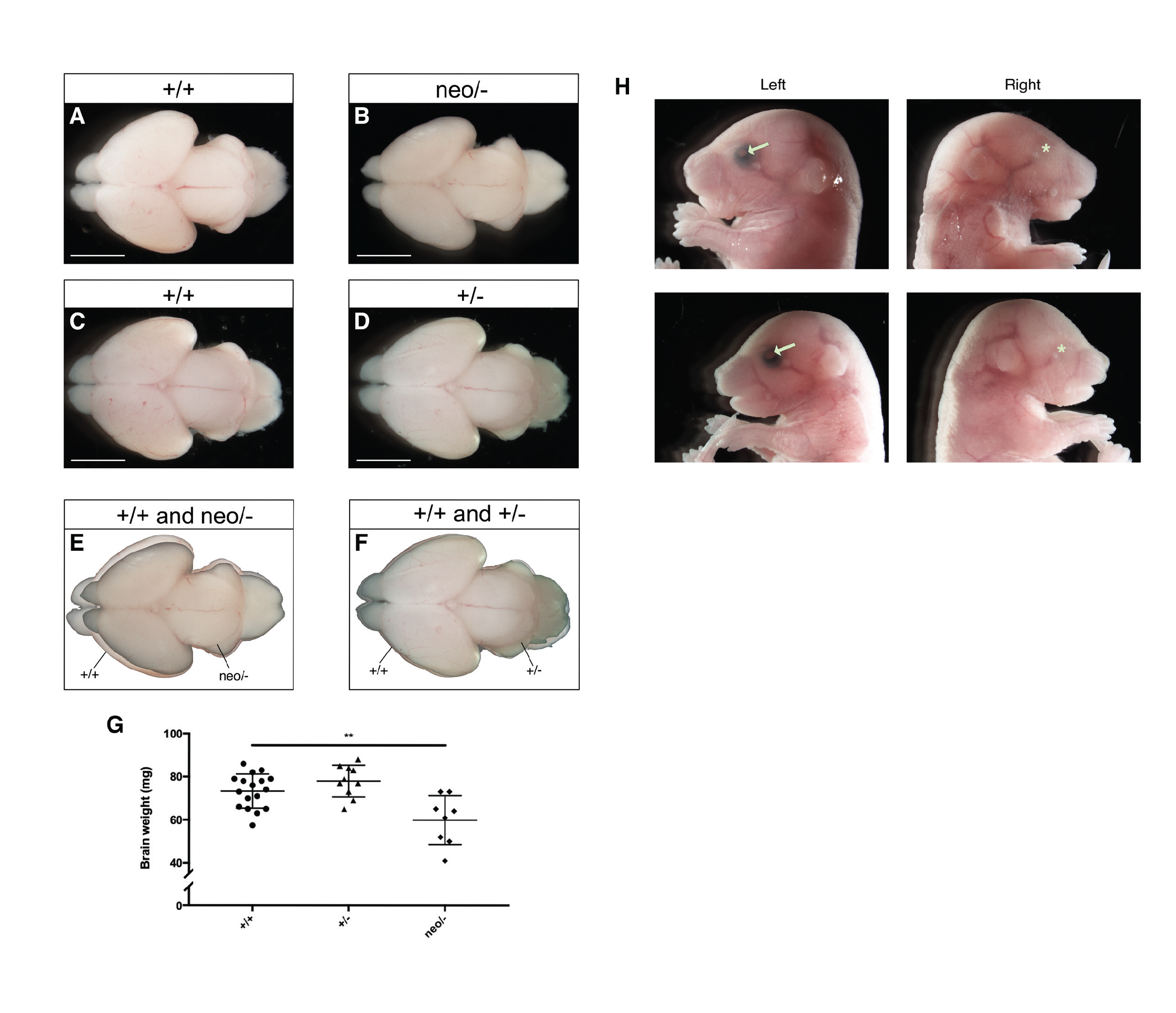


**Additional file 1: Figure S2. Brain hypoplasia and unilateral anopthalmia in severe *Chd8* hypomorphic mice**

A-D) Representative wholemount images of brains from E18.5 severe *Chd8* hypomorph (neo/-) and *Chd8* heterozygous (+/-) embryos, with corresponding wildtype (+/+) littermate controls (A for B and C for D).

E) Overlay of an E18.5 *Chd8^neo/-^* brain on to a littermate control brain. Note the smaller brain of the hypomorph.

F) Overlay of an E18.5 *Chd8^+/-^* brain on to a littermate control brain.

G) Absolute brain weights of wildtype control (+/+), *Chd8^+/-^* (+/-)*,* and *Chd8^neo/-^* (neo/-) brains*.* Note the significantly smaller brain weights in *Chd8^neo/-^* mice compared to controls, and the trend towards heavier brains in *Chd8^+/-^* embryos. n = 17 (+/+), n= 10 (+/-), n=8 (neo/-). Values plotted are mean ± SD. Scale bar = 2mm.

H) Lateral view of heads of E18.5 *Chd8^neo/-^* embryos with unilateral anopthalmia. White arrowheads indicate the presence of one eye on the left side of the head, whereas on the right side no apparent eye developed in the presumptive eye socket region. This was observed in two out of four *Chd8^neo/-^* embryos examined for this phenotype, each from a different pregnancy.

**
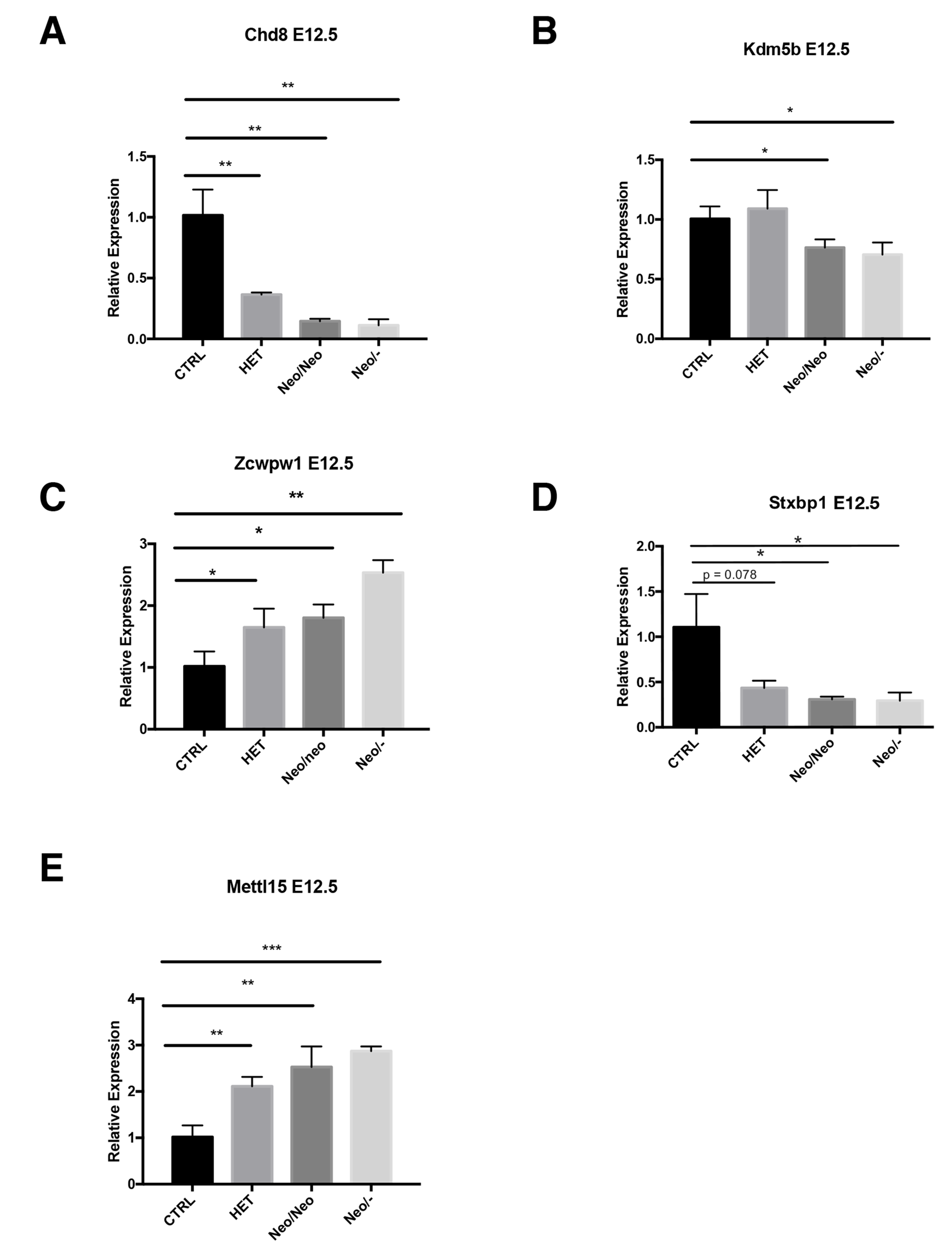
**

**Additional file 1: Figure S3. qRT-PCR validation of differential gene expression in E12.5 neocortex.**

A-E) Relative expression levels for a selection of DEGs detected in the E12.5 RNA-seq as determined by qRT-PCR. cDNA for qRT-PCRs was reverse transcribed from total RNA extracted from E12.5 neocortex for the genotypes shown. Relative expression levels were calculated using the 2^-∆∆CT^ method and *Canx*, *Sdha* and/or *Ywhaz* were used as endogenous control genes. (n = 3 per condition, Mean±SD, *p<0.05, **p<0.01, student’s t-test).

**
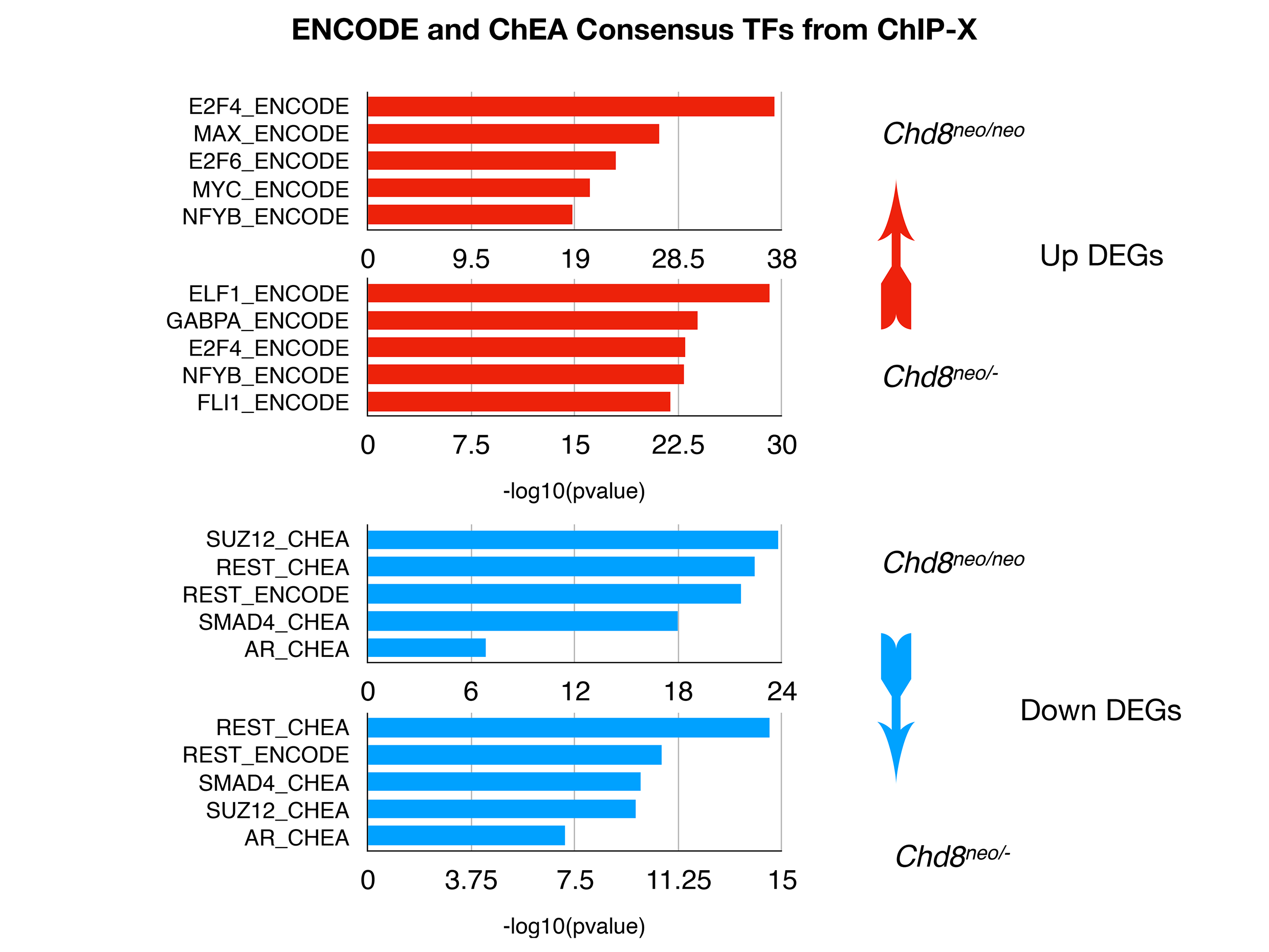
**

**Additional file 1: Figure S4. Analysis of transcription factor enrichment using Enrichr**

Putative regulatory transcription factors were determined with Enrichr using the “ENCODE and ChEA Consensus TFs from ChIP-X” database with all upregulated DEGs (top red panels) and downregulated DEGs (bottom blue panels) detected below a 0.05 FDR. The top 5 most significant hits are shown.

**
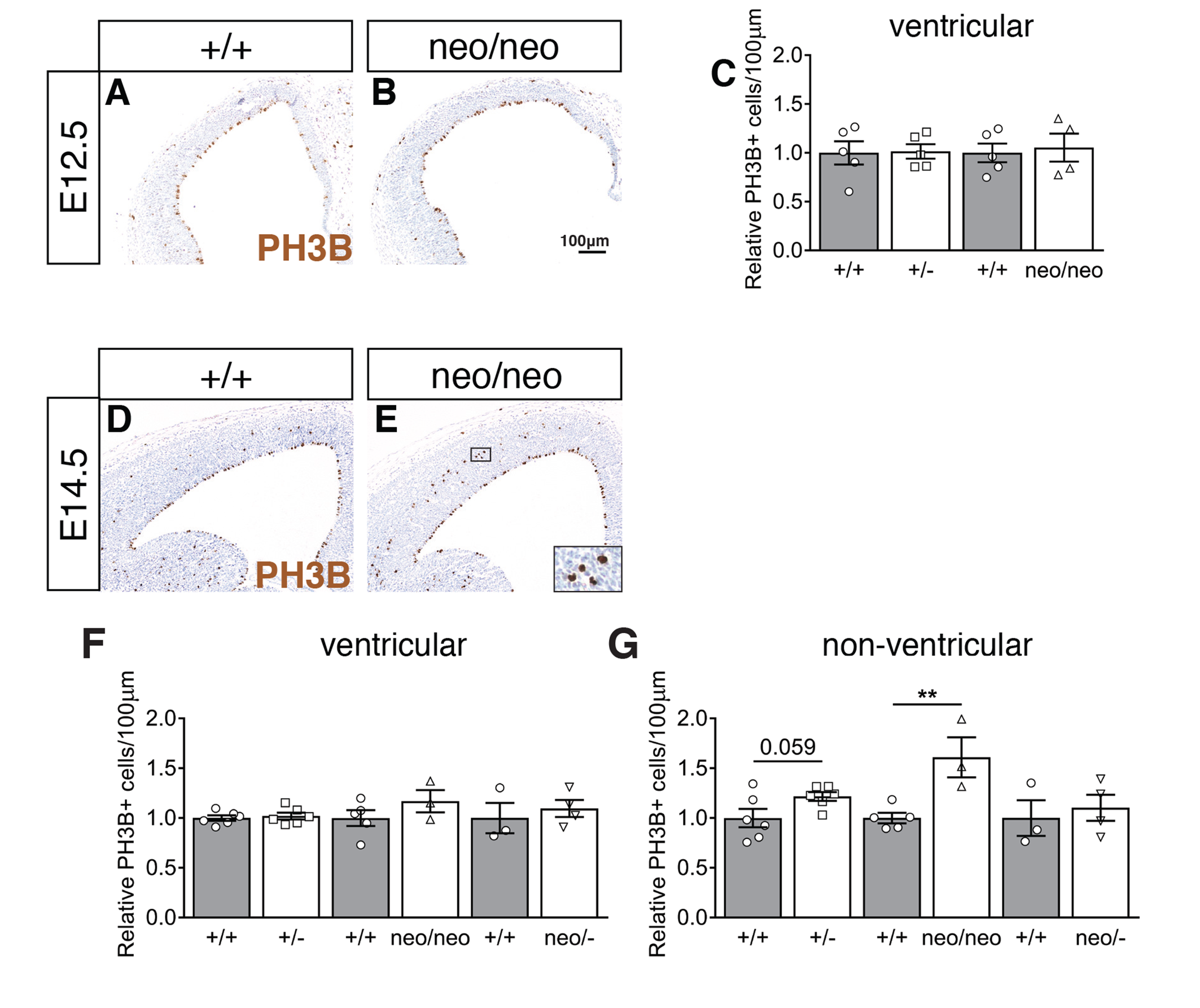
**

**Additional file 1: Figure S5. Non-ventricular progenitor proliferation is increased at E14.5 in *Chd8^neo/neo^* embryos.**

A,B) Immunostaining to detect PH3B+ nuclei (brown) in coronal sections through the telencephalon of E12.5 embryos of indicated genotypes. Scale bar = 100µm.

C) Quantification of PH3B+ cells per 100µm of ventricular zone in E12.5 embryos (+/+, n=5; +/-, n=5; +/+, n=5; neo/neo, n=4; Mean±SEM, student’s t-test).

D,E,F,G) Immunostaining to detect PH3B+ nuclei (brown) in coronal sections through the telencephalon of E14.5 embryos of indicated genotypes. Inset shows high magnification view of boxed area. Scale bar = 100µm.

F,G) Quantification of PH3B+ cells per 100µm in ventricular (F) or non-ventricular (G) areas of neocortex in E14.5 embryos (+/+, n=6; +/-, n=6; +/+, n=5; neo/neo, n=3; +/+, n=3; neo/-, n=4; Mean±SEM, **p<0.01, ANOVA followed by Tukey’s multiple comparisons test). Mutant and control samples for these experiment were obtained from 2-3 separate litters.

**
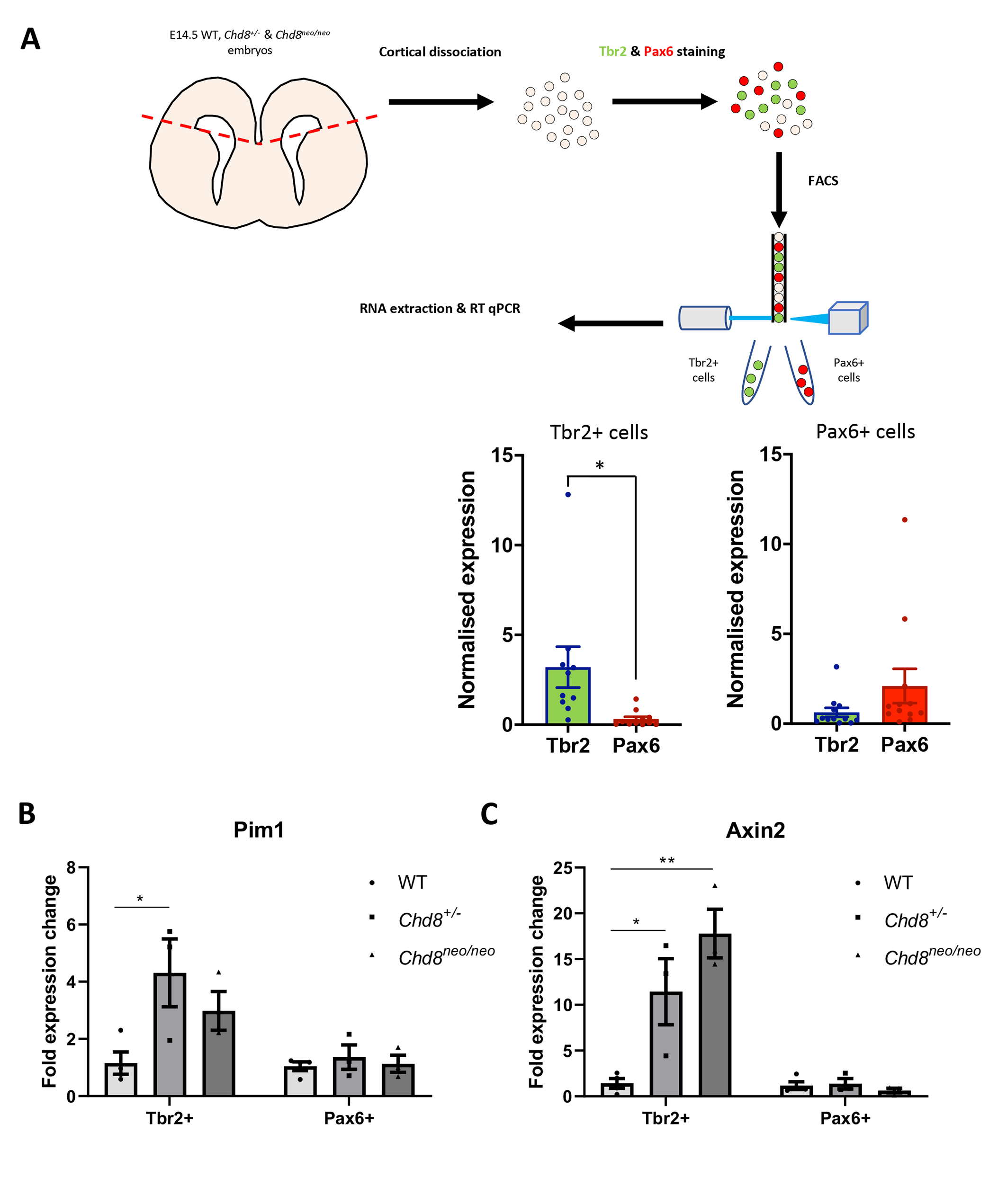
**

**Additional file 1: Figure S6. Gene expression in TBR2+ and PAX6+ neuronal progenitors in *Chd8* mutant embryos.**

A) Schematic of FACS/RT-PCR workflow. The neocortex was dissected from E14.5 embryonic brain and dissociated into single cell suspension. Cells were stained for TBR2 (AlexaFluor 488) and PAX6 (PE) and TBR2+ and PAX6+ populations isolated by FACS. RNA was extracted from samples, reverse transcribed to cDNA and quantitative PCR was performed. qRT-PCR analysis confirms enriched expression of *Tbr2* in Tbr2+ cells and enriched expression of *Pax6* in Pax6+ cells, although the latter is not statistically significant, probably due to low Pax6 expression in early TBR2+ progenitors. B,C) Fold expression changes in *Pim1* (B) and *Axin2* (C) in *Chd8^+/-^* and *Chd8^neo/neo^* cells compared to WT cells are shown (n=3 for each condition). Mean±SEM, *p<0.05, **p<0.01, unpaired student’s t-test.

**
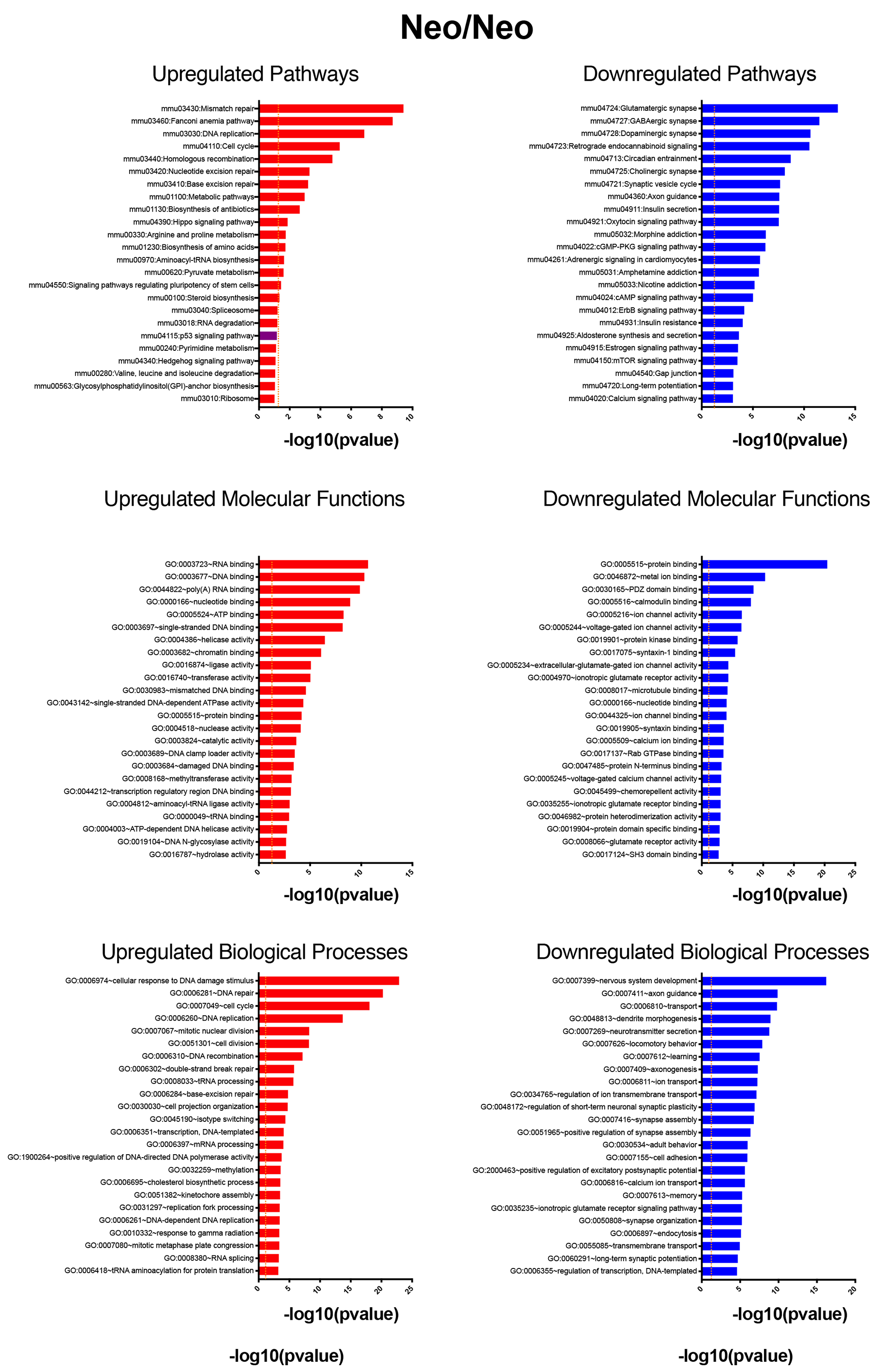
**

**Additional file 1: Figure S7. Functional Enrichment Analysis of differentially expressed genes (DEGs) in neo/neo embryos.**

DEGs (FDR <0.05) were subjected to KEGG pathway enrichment analysis (Top Panels), and screened for Gene Ontology terms in Molecular Function (Middle Panels) and Biological Process (Bottom Panels) categories using the DAVID knowledgebase. In each category, the 25 most significant terms are shown and the p53 pathway indicated in purple.

**
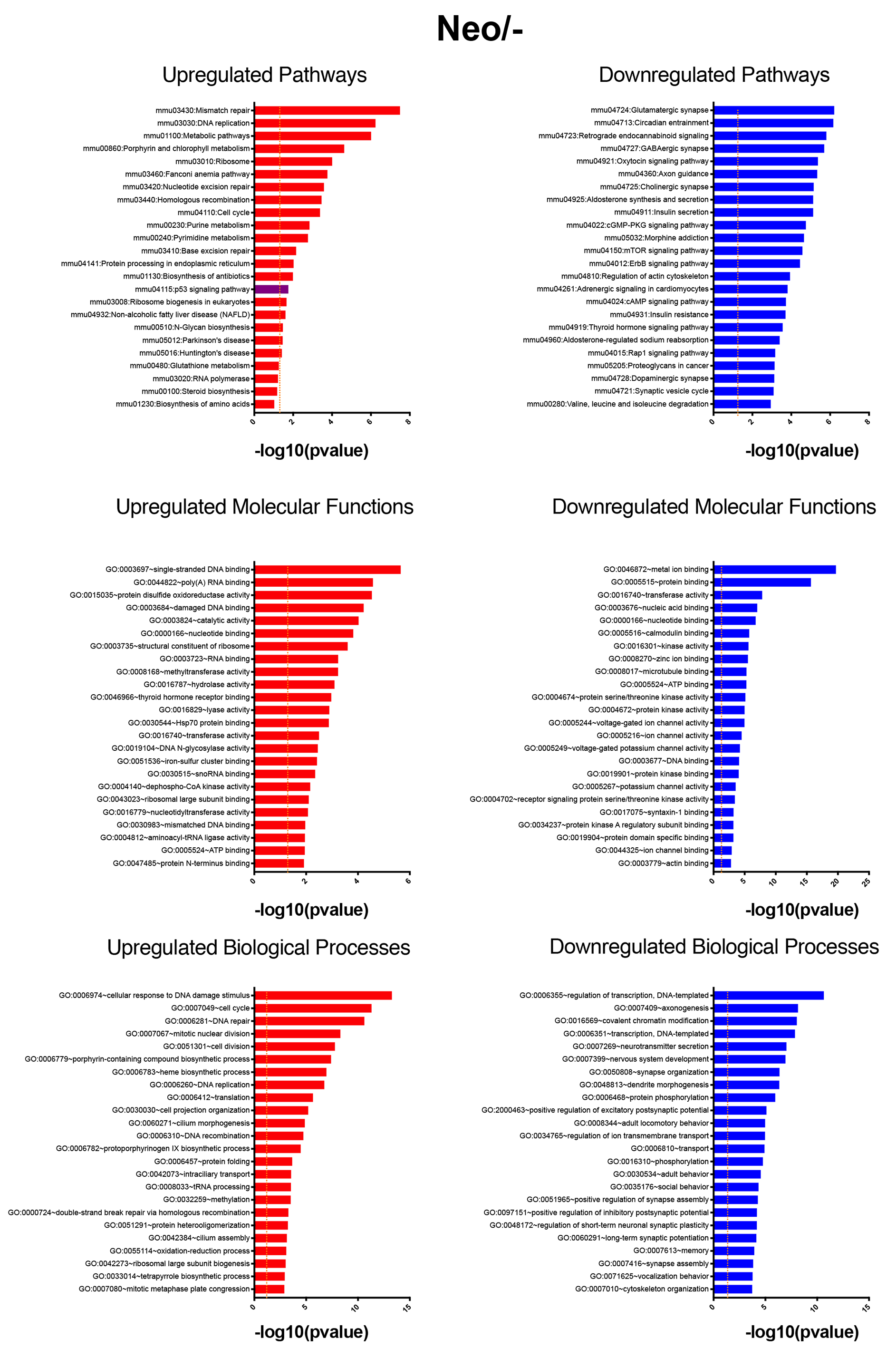
**

**Additional file 1: Figure S8. Functional Enrichment Analysis of differentially expressed genes (DEGs) in neo/- embryos.**

DEGs (FDR <0.05) were subjected to KEGG pathway enrichment analysis (Top Panels), screened for Gene Ontology terms in Molecular Function (Middle Panels) and Biological Process (Bottom Panels) categories using the DAVID knowledgebase. In each category, the 25 most significant terms are shown and the p53 pathway indicated in purple.

**
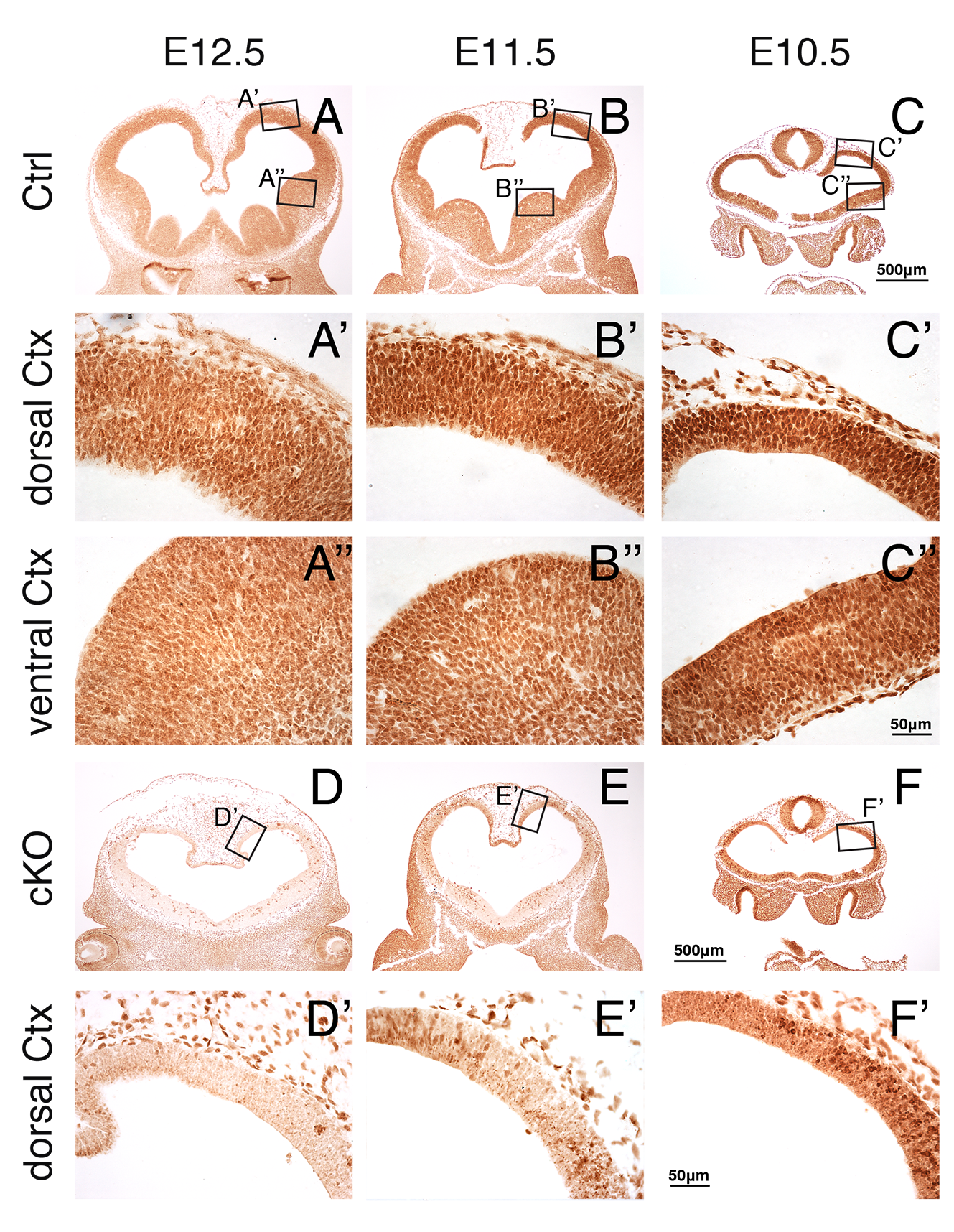
**

**Additional file 1: Figure S9. CHD8 is highly expressed during mid-embryonic stages and efficiently removed by recombination with *Sox1-Cre*.**

A-C’’) Immunostaining of E12.5, E11.5 and E10.5 brain sections with an anti-CHD8 antibody. Higher magnification images of dorsal cortex (boxed areas) are shown in A’-C”. Note the presence of nuclear CHD8 protein throughout the pallium and subpallium at E12.5 – E10.5 and higher levels of CHD8 immuno-staining in dorsal (A’-C’) compared to ventral pallium (A’’-C’’).

D-F’) Conditional pan-neuronal deletion of *Chd8* results in widespread loss of CHD8 protein in all brain structures. Higher magnification images of dorsal cortex (boxed areas) are shown in D’-F’. Note the loss of CHD8 immunostaining in the neural tube, with unchanged expression in other tissues. The data is representative of 3 control and 3 cKO embryos of each stage. Scale bars: A-F: 500μm, A’-F’, A’’-C’’: 50μm

**
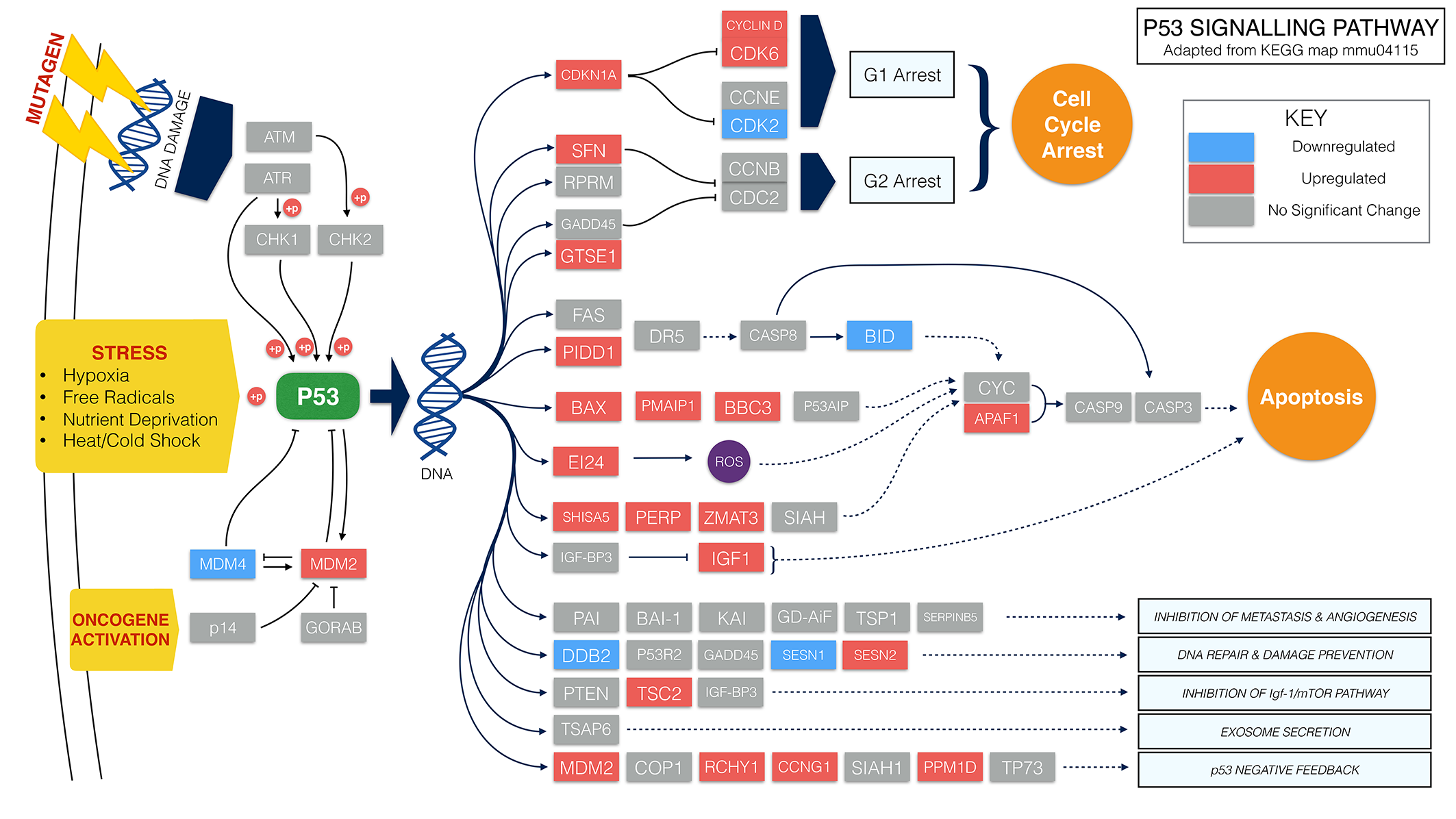
**

**Additional file 1: Figure S10. Diagram of the p53 pathway, with differentially expressed genes in the cKO highlighted.**

KEGG pathway mapping using DAVID of all differentially expressed genes revealed perturbed expression of 27 components of the p53 signalling pathway. Upregulated genes are boxed in red, downregulated genes in blue, and unchanged genes in grey. Solid lines indicate direct interactions whereas broken lines show indirect effects. *Chd8* depletion resulted in more upregulated (22 genes) than downregulated (5 genes) p53 pathway components. The expression of p53 itself (green) was not significantly changed. This figure is adapted from KEGG map mmu04115 (53).

**Additional file 1: Table S5: Primer sequences**

**qRT-PCR primer sequences:**

| **Gene** | **Forward Primer** | **Reverse Primer** |
| --- | --- | --- |
| **Atr** | GAATGGGTGAACAATACTGCTGG | TTTGGTAGCATACACTGGCGA |
| **Atm** | TCTGTCCAGCAAAATCTCAAGG | CCCATGTAACAATAGCAGCCAA |
| **Mdm2** | TGTCTGTGTCTACCGAGGGTG | TCCAACGGACTTTAACAACTTCA |
| **Ccng1** | AGTCGGCCCATGATAATGGC | GTCCAACACACCCAAGATGC |
| **Trp53** | GGGGAGGAGCCAGGCCATCA | CCGCGCCATGGCCATCTACA |
| **Pmaip1** | TGGAGTGCACCGGACATAAC | AATCCTCCGGAGTTGAGCAC |
| **Chd8** | CAGAGGAGGAGGGTGAAAAGAAAC | GAGTTGTCAGACGATGTGTTACGC |
| **Cdkn1a** | TTGGAGTCAGGCGCAGATC | GACCGAAGAGACAACGGCA |
| **Kdm5b** | AAGCCAAGCTCTGTTCAGCAA | GAAGGCAATCGTTCTTCTCACT |
| **Zcwpw1** | GATGAAGAACCGGGCATTGTT | GGCCTAGCTTAGATGTCCCCA |
| **Stxbp1** | CATGAGAGCCATTGTCCCCA | AGTGCTTTGTATCCAGCTTGTC |
| **Gapdh** | AGGTCGGTGTGAACGGATTTG | TGTAGACCATGTAGTTGAGGTCA |
| **Ywhaz** | GAAAAGTTCTTGATCCCCAATGC | TGTGACTGGTCCACAATTCCTT |
| ***Pax6*** | GCAGATGCAAAAGTCCAGGTG | CAGGTTGCGAAGAACTCTGTTT |
| ***Tbr2*** | CCTACCAAAACACGGATATCACCC | TGTCATTTTCTGAAGCCGTGTA |
| **Axin2** | CCCCACCTTGAATGAAGAAGAGG | GCTGGATAACTCGCTGTCGTTG |
| **Pim1** | GCGGCGAAATCAAACTCATC | TGGTAGCGAATCCACTCTGG |
| **Otx1** | TCTAACGTCCAATGCGGCTG | GGTTGTTTGGAGGCGCAAAG |
| **Ier2** | TGACTCTGTCGGTATGGAAGAT | ACCTTGGCTGAGAGGTAGACC |
| ***Cdkn1b*** | TCAAACGTGAGAGTGTCTAACG | CCGGGCCGAAGAGATTTCTG |

**Genotyping primer sequences:**

| **Gene** | **Forward Primer** | **Reverse Primer** |
| --- | --- | --- |
| **Chd8flox/ Chd8neo** | GCCGAGGGGATGAGGATATTTAGG | GGTACATATGCCTTAAAAATCAGGCCCAG |
| **Chd8null** | CCCACATCAAGTGGCTGTAA | GGTAGGGAAGCAGTGTCCAG |
| **p53flox** | GGTTAAACCCAGCTTGACCA | GGAGGCAGAGACAGTTGGAG |
| **Cre** | CCTGGAAAATGCTTCTGTCCG | CAGGGTGTTATAAGCAATCCC |

**Neo allele splice variant primer sequences:**

| **Primer name** | **Sequence** |
| --- | --- |
| **F** | CAGAGGAGGAGGGTGAAAAGAAAC |
| **R** | GAGTTGTCAGACGATGTGTTACGC |
| **neo** | GATATTGCTGAAGAGCTTGGC |
